# The *Cis*-Regulatory Code for *Kelch-like* 21/30 Specific Expression in *Ciona robusta* Sensory Organs

**DOI:** 10.3389/fcell.2020.569601

**Published:** 2020-09-11

**Authors:** Ugo Coppola, Ashwani Kumar Kamal, Alberto Stolfi, Filomena Ristoratore

**Affiliations:** ^1^Biology and Evolution of Marine Organisms, Stazione Zoologica Anton Dohrn Napoli, Naples, Italy; ^2^School of Biological Sciences, Georgia Institute of Technology, Atlanta, GA, United States

**Keywords:** tunicates, otolith, *Mitf*, *Klhl* family evolution, pigment cells, CRISPR/Cas9, *cis*-regulatory regions

## Abstract

The tunicate *Ciona robusta* is an emerging model system to study the evolution of the nervous system. Due to their small embryos and compact genomes, tunicates, like *Ciona robusta*, have great potential to comprehend genetic circuitry underlying cell specific gene repertoire, among different neuronal cells. Their simple larvae possess a sensory vesicle comprising two pigmented sensory organs, the ocellus and the otolith. We focused here on *Klhl21/30*, a gene belonging to *Kelch* family, that, in *Ciona robusta*, starts to be expressed in pigmented cell precursors, becoming specifically maintained in the otolith precursor during embryogenesis. Evolutionary analyses demonstrated the conservation of *Klhl21/30* in all the chordates. *Cis*-regulatory analyses and CRISPR/Cas9 mutagenesis of potential upstream factors, revealed that *Klhl21/30* expression is controlled by the combined action of three transcription factors, Mitf, Dmrt, and Msx, which are downstream of FGF signaling. The central role of Mitf is consistent with its function as a fundamental regulator of vertebrate pigment cell development. Moreover, our results unraveled a new function for Dmrt and Msx as transcriptional co-activators in the context of the *Ciona* otolith.

## Introduction

Understanding the developmental logics that orchestrate specific gene expression inside the nervous system represents a fascinating challenge in cell and developmental biology. However, the identification of the molecular processes underlying cell specific expression among different neuronal cells is very difficult using vertebrate models, due to the relative complexity of vertebrate embryos and genomes, as well as the numbers of genes involved.

Within the chordates, the tunicate subphylum is the sister group of vertebrates, forming with them the clade Olfactores (Delsuc et al., 2006). Due to their small, invariant embryos and compact genomes (Berná and Alvarez-Valin, 2014), tunicates have great potential to help to uncover the genetic circuitry regulating chordate-specific mechanisms of neural development. The larva of the tunicate *Ciona robusta* possesses two distinct pigmented sensory organs, the otolith and the ocellus, contained in the anterior sensory vesicle, a structure evolutionarily related to the forebrain of vertebrates (Moret et al., 2005; Dufour et al., 2006). The ocellus is formed by 30 photoreceptors, three lens cells and one melanized cup-shaped cell (Horie et al., 2005). Due to its association with photoreceptors, the ocellus has been compared to the vertebrate eye (Kusakabe et al., 2001; Nakagawa et al., 2002; Nakashima et al., 2003; D’Aniello et al., 2006). In contrast, the otolith is a single spherical cell containing melanin granules and attached to the sensory vesicle floor through a tight stalk (Dilly, 1962, 1969). It has been proposed that the displacement of the otolith within the fluid-filled sensory vesicle lumen stimulates a putative mechanosensory antenna cell in the adjacent brain, thus playing a key role in negative geotropism and gravitational orientation (Tsuda et al., 2003; Sakurai et al., 2004). Supporting this model, an ammonium channel regulates the sensory vesicle fluid composition and otolith functioning (Marino et al., 2007). In addition, the pigmentation of these sensory organs is fundamental for normal photo- and geotactic behaviors in the close sibling species *C. savignyi* (Jiang, 2005).

During embryonic development, ocellus and otolith pigment cells derive from a pair of left/right pigment cell precursors (PCP) in the neural plate that intercalate at the dorsal midline of the neural tube during neurulation (Cole and Meinertzhagen, 2004). Later, during neural tube closure, PCPs divide twice and give rise to a total of 8 cells at early tailbud stage, which express melanogenic genes belonging to the *Tyrosinase (Tyr)* family (Tief et al., 1996; Caracciolo et al., 1997; Esposito et al., 2012; Haupaix et al., 2014; Racioppi et al., 2014, 2017). The loss of *Tyr* function in *Ciona* results in pigment-free larvae (Sordino et al., 2008; Crocetta et al., 2015), whereas some tunicate species (e.g., *Molgula occulta*) have lost *Tyr* family genes and lack melanin pigmentation altogether (Racioppi et al., 2017).

The majority of the transcription factors and cell signaling molecules implicated in otolith and ocellus differentiation are expressed in the whole PCP lineage, even though only two cells will become pigmented. A FGF-dependent transcriptional code for the formation of *Ciona* PCPs was recently partially elucidated (Racioppi et al., 2014). Among the two pigmented cells the anterior-posterior order of intercalation orchestrates ocellus *versus* otolith pigment cell determination: the anterior cell always becomes the otolith while the posterior cell always gives rise to the ocellus pigment cell. These mechanisms, which are still not well studied, involve a Wnt and FoxD-mediated suppression of Pax3/7-dependent activation of the *Microphthalmia-associated transcription factor* gene (*Mitf*) (Abitua et al., 2012).

Here, we focused on the transcriptional control of *Klhl21/30*, a gene that we found to be specifically expressed in the ocellus and otolith precursors, during embryogenesis. *Klhl21/30* belongs to the *Kelch-like* (*Klhl*) family of genes, encoding for proteins characterized by the presence of multiple Kelch motifs, which are evolutionarily conserved, but poorly characterized, short domains implicated in protein-protein interactions (Adams et al., 2000). In mammalian cells, Klhl21 is thought to be involved in E3-ubiquitination during cytokinesis and regulation of cortical dynamics (Maerki et al., 2009; Courtheoux et al., 2016) and is implicated in diverse types of carcinoma (Shi et al., 2016; Chen et al., 2018). We provide the most updated evolutionary reconstruction of *Klhl* family, demonstrating that *Klhl21/30* is ultra-conserved in chordates. This gene has dynamic expression pattern in *Ciona* PCPs, becoming restricted to the otolith during embryogenesis. *Klhl21/30* transcription is governed by a complex regulatory code, in fact, we have identified the minimal key *cis*-regulatory element able to drive *Klhl21/30* expression in the *C. robusta* otolith, containing functional binding sites for the transcription factors Mitf, Msx, and Dmrt. Using tissue-specific CRISPR/Cas9-mediated gene knockouts, we found that Mitf is central to *Klhl21/30* expression in the otolith, as it is for many pigmentation markers in the pigment cells of vertebrates (Levy et al., 2006), with Msx and Dmrt acting as co-activators.

## Results

### *Kelch-like* Gene Family Evolution in Chordates

A lineage-specific transcription profiling of genes downstream of fibroblast growth factor signaling (FGF), was used to find novel players involved in pigment cell formation (Racioppi et al., 2014). Among these, we found a *Kelch-like* (*Klhl*) gene family member (Kyoto Hoya gene model KH.L84.23), which exhibited similar expression values of known PCP markers, including *Tyr*, *Tyrp.a* and *Rab32/38* and that, by reciprocal BLASTs, is similar to vertebrate *Kelch-like 21* (*Klhl21*) and *Kelch-like 30* (*Klhl30*).

**FIGURE 1:**
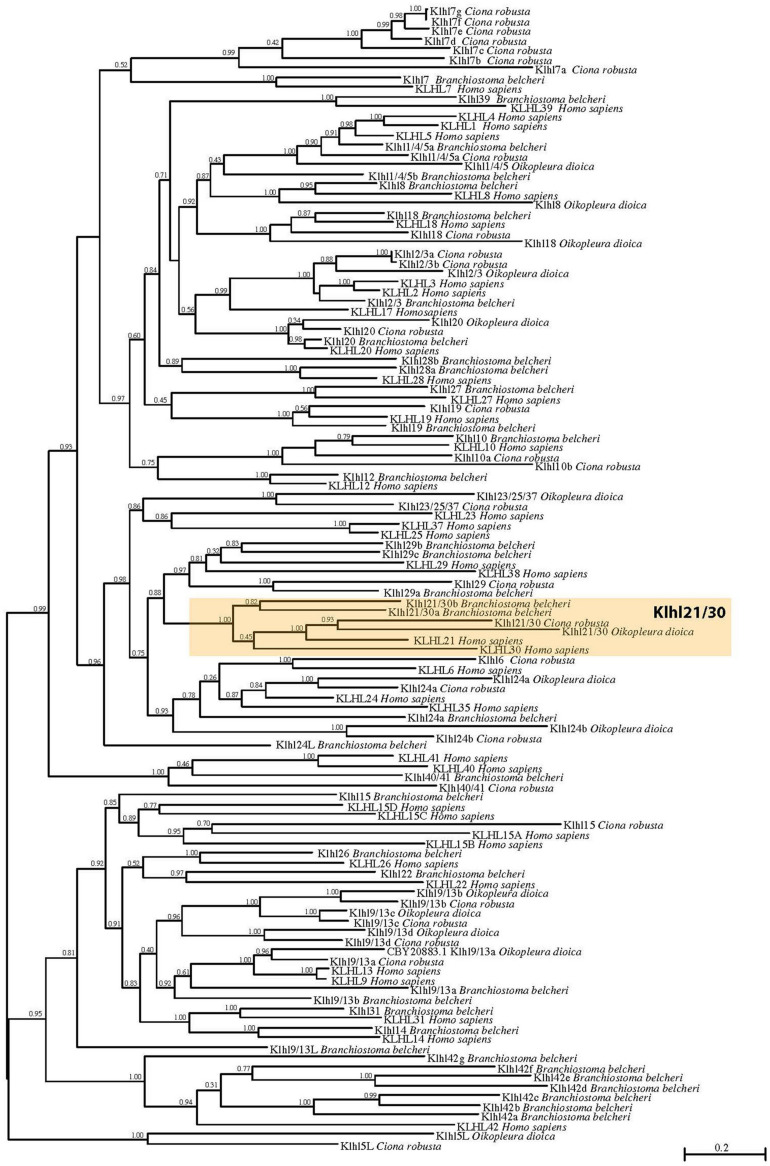
Phylogenetic tree of Kelch-like proteins in chordates. Maximum likelihood (ML) reconstruction of 118 Kelch-like members from four key chordate species: amphioxus *Branchiostoma belcheri*, larvacean *Oikopleura dioica*, ascidian *Ciona robusta*, human *Homo sapiens*. The phylogeny demonstrates the existence of a robust Klhl21/30 subfamily (orange box). Numbers at the branches indicate replicates obtained using the ML estimation method.

To shed light on the evolutionary origins of the *Klhl21/30* gene and to gain insights into the poorly studied *Klhl* family in metazoans, we performed an evolutionary analysis of these proteins in chordates. First, we analyzed their domain organization in PROSITE (de Castro et al., 2006) and Ensembl databases. This revealed the presence of one BTB/POZ domain, one BACK domain and five Kelch repeats (Supplementary Figure S1) in both *C. robusta* Klhl21/30 and *Homo sapiens* KLHL21 (Dhanoa et al., 2013). To confirm the orthology of C. robusta *Klhl21/30* (KH.L84.23) and study the evolution of *Kelch-like* genes in chordates, we performed a phylogenetic survey employing a manually curated database (Supplementary Table S1). Our phylogenetic reconstruction included 118 Kelch-like protein sequences from the cephalochordate amphioxus *Branchiostoma belcheri*, tunicates *Oikopleura dioica* and *Ciona robusta*, and human *Homo sapiens* as representative of vertebrates (Figure 1). Sequences with high degree of molecular divergence have been excluded from phylogeny and listed in the Supplementary Table S2. In total, we identified 25 Klhl subfamilies, conserved throughout chordate evolution from cephalochordates to vertebrates, with a unique exception (*Klhl5L*) that is tunicate-specific (Figure 1). Genome search and phylogenetic tree defined the complete Kelch-like toolkit of *B. belcheri* (37), *C. robusta* (32), *O. dioica* (14), *H. sapiens* (42): this confirms the previous survey performed in human (Dhanoa et al., 2013) and represents the first step into understanding Kelch-like evolution in all three chordate subphyla. Though the number of *Kelch-like* genes in the amphioxus genome has been shaped by various amphioxus-specific duplications (as in the case of *Klhl42* duplications), our survey is coherent with relative genomic stasis of this slow-evolving branch (Putnam et al., 2008). In contrast, *C. robusta* retained 32 *Klhl* genes and 16 subfamilies, whilst *O. dioica* maintained 14 genes and 10 subfamilies. This is coherent to the tendency of the fast-evolving *Oikopleura dioica* to lose large portions of gene families (Berná and Alvarez-Valin, 2014; Albalat and Cañestro, 2016; Martí-Solans et al., 2016; Coppola et al., 2019). Otherwise, we also detected different tunicate-specific duplications of certain *Kelch-like* genes in either species (Figure 1).

In particular, our phylogenetic tree clustered Ciona KH.L84.23 with one *O. dioica* protein, two *B. belcheri* proteins (deriving from a local duplication) and *H. sapiens* KLHL21 and KLHL30, forming a protein class with robust support that we named Klhl21/30 (orange box; Figure 1). The presence of two related genes in human speaks in favor of a common origin for vertebrate *Klhl21* and *Klhl30*, which could have derived from a local duplication or a whole-genome duplication event in the vertebrate ancestor (Abi-Rached et al., 2002; Dehal and Boore, 2005). In contrast, invertebrate chordates possess a single *Klhl21/30* gene, similar to other invertebrates such as the nematode *Caenorhabditis elegans* and the sea urchin *Strongylocentrotus purpuratus* (data not shown). Remarkably, *Klhl21/30* is one of the few *Kelch-like* genes not lost by *O. dioica* and therefore could be functionally relevant for conserved developmental processes. Moreover, we analyzed the *Klhl21/30* locus in several available tunicate genomes, finding high degree of local synteny surrounding *Klhl21/30* in *C. robusta*, *C. savignyi*, *Phallusia mammillata*, and *Halocynthia roretzi* (Supplementary Figure S2). Our survey prompted us to describe an ancestral cluster of 11 genes in tunicates, nearly all conserved between *C. robusta* and *P. mammillata*. Interestingly, all surveyed species exhibited linkage between *Klhl21/30* and *Nph4* (*nephrocystin-4*) genes, which is also observed in human between *KLHL21* and *NPH4*. Conversely, this linkage has been lost for *KLHL30* (Supplementary Figure S3). The comparison of *Klhl21* and *Klhl30* genome environment of species as coelacanth *Latimeria chalumnae*, spotted gar *Lepisosteus oculatus*, frog *Xenopus tropicalis* and human, demonstrated the conservation of flanking genes during gnathostome evolution (Supplementary Figure S3). Moreover, the presence of orthologous genes on both the surveyed chromosomal regions, strongly indicated *Klhl21* and *Klhl30* as paralogs deriving from one of the events of whole-genome duplication (WGDs) occurred at the root of vertebrate radiation (Dehal and Boore, 2005).

In sum, we have reported here the first evolutionary study dedicated to *Kelch-like* family in chordates, focused on the seemingly indispensable *Klhl21/30* subfamily, which was present in all chordate species examined.

### *Klhl21/30* Dynamic Expression in Otolith

**FIGURE 2:**
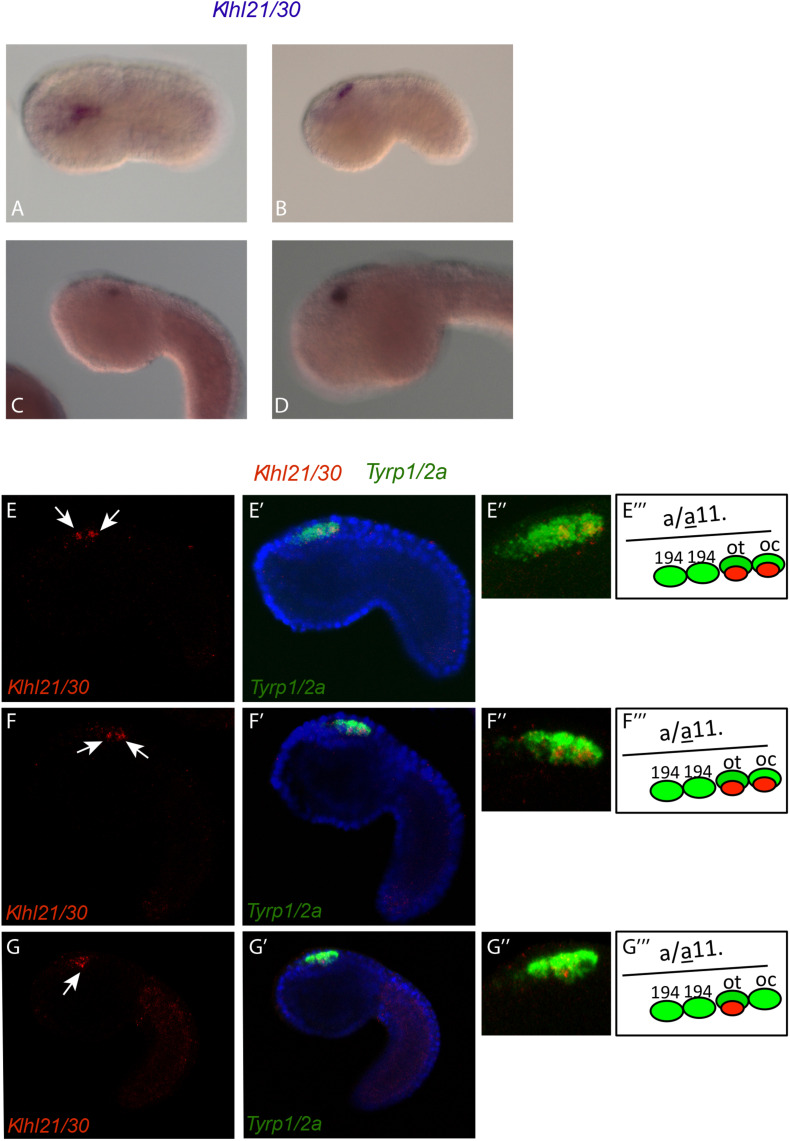
Expression of *Klhl21/30* during *Ciona robusta* embryogenesis. *Klhl21/30* expression was detected by *in situ* hybridization in the *C. robusta* pigment cell lineage at initial tailbud **(A)**, early tailbud **(B)**, middle tailbud **(C)**, late tailbud **(D)**. Double WISH with *Tyrp1/2a* that colocalizes precisely *Klhl21/30* among PCPs. **(E–F”’)** Early tailbud I **(E–E”)** and early tailbud II **(F–F”)** stages, white arrow indicates the two cells expressing *Klhl21/30* that colocalizes with the posterior two *Tyrp1/2a* positive cells. **(G–G”’)** Middle tailbud stage. One white arrow indicates the sole cell expressing *Klhl21/30* at this stage that colocalizes with otolith precursor as shown in **(G–G”’)**.

The analysis of the spatio-temporal expression pattern of *Klhl21/30*, by whole mount *in situ* hybridization (WISH) in embryos at different developmental stages (Figures 2A–D), showed that during *C. robusta* embryogenesis, *Klhl21/30* is expressed from initial tailbud stage onward in the pigment cell lineage (Figures 2A–D). In particular, at initial tailbud stage it is expressed in two cells (Figures 2A,B), possibly the ones originating the otolith and ocellus pigmented cells of the larva. Later in development, from the middle tailbud stage onward, *Klhl21/30* transcript became restricted specifically to one cell (Figures 2C,D). Double WISH using the pigment cell marker, *Tyrp1/2a*, was performed to better define PCP-specific expression. Detailed and thorough analysis of the expression domain proved that *Klhl21/*30 is expressed specifically in the a11.193 pair, i.e. the most posterior cells marked by *Tyrp1/2a* (Haupaix et al., 2014; Racioppi et al., 2014) (white arrow in Figures 2E–F”’) later, starting from middle tailbud stage, the *Klhl21/30* expression became restricted to the otolith precursor (white arrow in Figures 2G–G”’), while it disappears from the ocellus pigment cell precursor. Thus, *Klhl21/30* represents the earliest-expressed otolith specific gene described so far. Its expression is maintained in the otolith pigment cell precursor while it is downregulated in the ocellus pigment cell precursor, even before expression of the βγ*-crystallin*, which is expressed specifically in the otolith starting from the larval stage (Shimeld et al., 2005). *Klhl21/30* expression in PCPs is consistent with microarray data (Racioppi et al., 2014), in which *Klhl21/30* expression was observed to decrease between 8 and 12 hpf. Furthermore, its expression was shown to be dependent on the FGF signaling, which is required for PCPs specification. Coherently, *in situ* hybridizations performed using *Klhl21/30* probe on tailbud embryos electroporated with a construct, *Tyrp* > *dnFGFr*, able to drive a dominant-negative form of the FGF receptor in the PCPs (Squarzoni et al., 2011), showed a sharp decrease in the number of tailbud embryos expressing *Klhl21/30* in PCPs compared to control (Supplementary Figure S4). Altogether, these data identified *Klhl21/30* as the earliest marker of the *Ciona* otolith and the first *Klhl* member described in tunicates.

### *Cis*-Regulatory Analysis of *Klhl21/30* Expression

**FIGURE 3:**
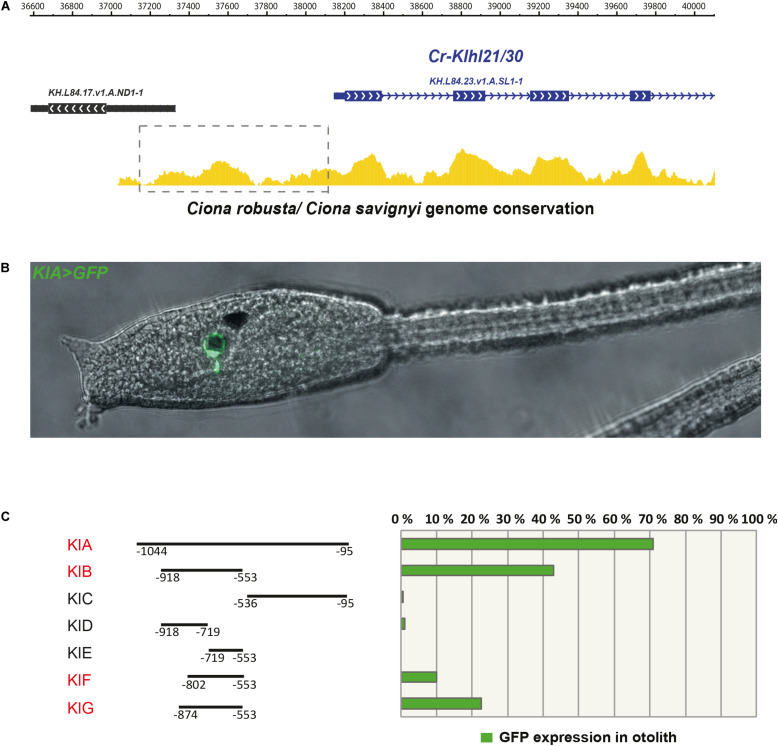
The *Klhl21/30 cis*-regulatory region. **(A)** Alignment of *Klhl21/30* locus between *Ciona robusta* and *Ciona savignyi* employing WASHU browser, with a dashed rectangle indicating the surveyed intergenic region. **(B)** Side view of larva embryo electroporated with *KlA* > *GFP* construct. **(C)** Percentage of larvae expressing GFP reporter in otolith with schematic representations of the dissected fragments and analysis by gene reporter assay (the fragments driving expression in otolith are highlighted in red); each bar represents the combined number of larvae counted during at least three trials; *n* > 150 embryos scored for transgene expression.

**FIGURE 4:**
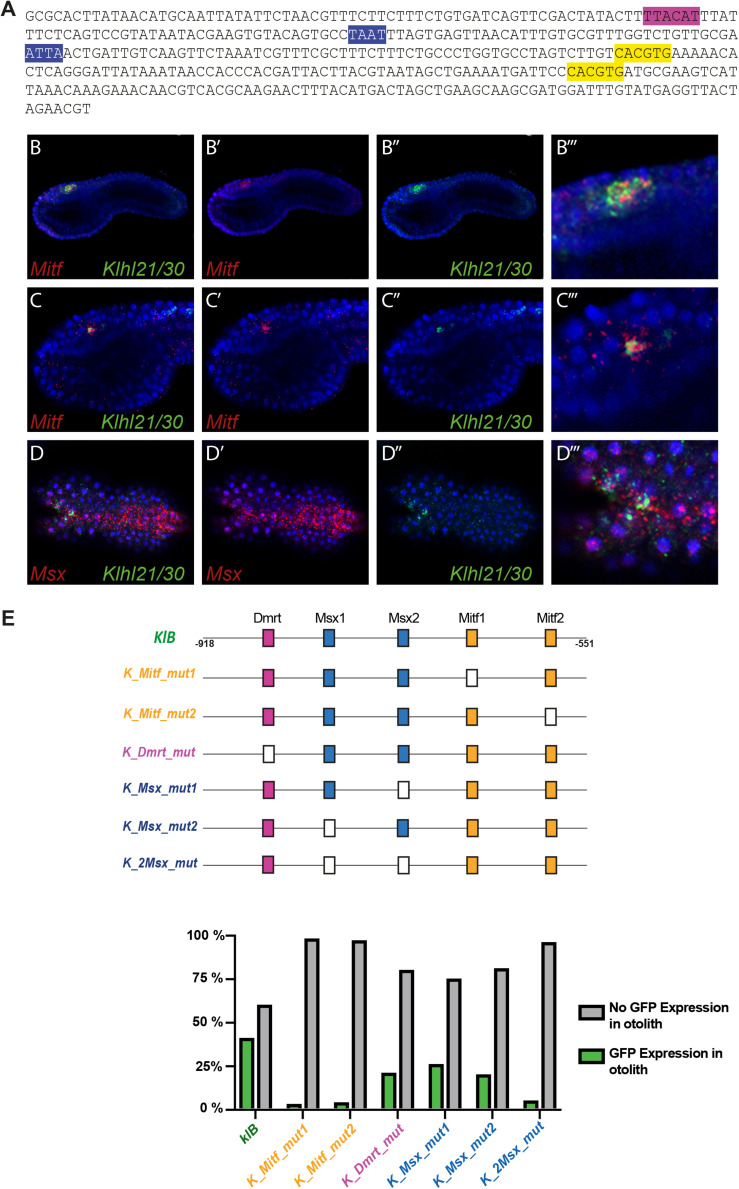
Regulatory logic underlying *Klhl21/30* expression. **(A)** *KlB* region with the transcription factor binding sites (TFBS) analyzed: Mitf (yellow-orange), Dmrt (violet), Msx (blue). **(B)** Co-expression in pigment cell precursors of *Klhl21/30* with *Mitf* at early tailbud **(B–B”’)** and middle tailbud **(C–C”’)**, and *Msx* **(D–D”’)**, respectively. **(E)** Mutational analysis with percentages of larvae expressing GFP, employing *KlB* > *GFP* as control; the TFBS are shown using rectangles with the same color code reported for the sequence, while the mutated sites are represented with white rectangles. Experiments were repeated at least three times and 200 embryos were counted each time; all the mutated constructs were significant versus the control *KlB* (Fisher exact test, *p* < 0.00001).

Since *Klhl21/30* was identified as a specific marker for PCPs as well as the earliest otolith marker in *Ciona robusta*, we sought to study its transcriptional regulation to understand the regulatory logics underlying differentiation of these structures (Figure 3). In order to discover the *cis*-regulatory element involved in the control of *Klhl21/30* expression, we took advantage of the genome conservation tracks in the ANISEED genome browser (Brozovic et al., 2018) to select a 949-bp non-coding region upstream *Klhl21/30* (“*KlA”*, −1044 to −95 from the start codon), a region presenting conserved peaks with the sibling species *C. savignyi* (Figure 3A). This *KlA* fragment was cloned in a vector containing a *GFP* reporter gene downstream of a human β*-globin* minimal promoter (Zeller et al., 2006). Once electroporated the *KlA* > *GFP* reporter plasmid into *Ciona* embryos (Corbo et al., 1997), we detected otolith-specific GFP fluorescence in 70% of larvae at stage 26 (Hotta et al., 2007; Figures 3B,C). By WISH using *GFP* probe, we registered transcription of *KlA* > *GFP* at the middle tailbud stage in one cell of the sensory vesicle, possibly corresponding otolith precursor (Supplementary Figure S5). This indicates that the absence of GFP fluorescence until the larval stage is due to a delay in GFP maturation and accumulation. Our reporter plasmid thus recapitulates the endogenous expression of *Klhl21/30* at least in the otolith, confirming the restriction of its expression to the otolith precursor in the late embryogenesis. To dissect the regulatory logics underlying the expression of *Klhl21/30*, we focused on two smaller, highly conserved regions within the *KlA* sequence: *KlB* (385 bp long, −918 to −553) and *KlC* (441 bp long, −536 to −95). While *KlC* did not drive GFP expression, 43% of larvae electroporated with *KlB* > *GFP* recapitulated the strong GFP signal in otolith, suggesting that *KlB* fragment retains the minimal regulatory information necessary to drive *Klhl21/30* expression (Figure 3C). When we divided *KlB* roughly into two halves, *KlD* (199 bp long, −918 to −719) and *KlE* (166 bp long, −719 to −553), neither was sufficient to drive GFP expression (Figure 3C). However, we found that two smaller fragments centered around *KlB*, which are *KlF* (257 bp long, −810 to −553) and *KlG* (321 bp long, −874 to −553), drove GFP expression in 10 and 22% of larvae, respectively (Figure 3C). These fragments therefore represent the minimal *cis*-regulatory elements sufficient to recapitulate *Klhl21/30* expression. We hypothesized that the *KlB* region contains transcription factors binding sites (TFBS) crucial for the sustained activation of *Klhl21/30* in pigment cell precursors. Therefore, we searched in this fragment putative TFBS using Genomatix software and CIS-BP database with *C. intestinalis* motifs (Weirauch et al., 2014) and these results have been compared to JASPAR database (Khan et al., 2018; Figure 4). Among several predicted binding sites, we focused our attention on two bHLH-binding motifs. In *Ciona* 44 *bHLH* genes have been found (Satou et al., 2003) and, for most of them, expression pattern during development has been described (ANISEED database). We selected Mitf as possible factor binding the bHLH sites in the *Klhl21/30* regulatory region (yellow in Figure 4A) because of involvement of *Mitf* in eye development and in the activation of melanogenic markers *Tyr* and *Tyrp/DCT* in vertebrates (Goding, 2000; Curran et al., 2010) and its specific expression in the pigment cell lineage of *Ciona* (Curran et al., 2010; Abitua et al., 2012) and *Halocynthia roretzi* (Yajima et al., 2003). We demonstrated that *Mitf* is co-expressed with *Klhl21/30* in both otolith and ocellus pigmented cells precursors at early tailbud stage (Figures 4B–B”’) and that both became restricted to otolith precursor starting from middle tailbud stage (Figures 4C–C”’). Previous experiments showed also that *Mitf* is downregulated upon perturbation of FGF signaling (Racioppi et al., 2014). To test the relevance of these two putative sites for Mitf (CACGTG), we mutated them individually in the *KlB* reporter, naming these mutant constructs *K_Mitf_mut1* and *K_Mitf_mut2* (Figure 4E). When each of these constructs was electroporated, the percentage of larvae showing GFP signal in the otolith was close to zero (2.5 and 3%, respectively, Figure 4E). These data strongly support these binding sites for Mitf as involved in *KlB* activity, leading to hypothesize that Mitf activates *Klhl21/30* in *Ciona robusta*.

We also identified a well-supported binding site in *KlB* for Dmrt (TTACAT, violet in Figure 4A), a transcription factor expressed in the early a-line neural plate (Wagner and Levine, 2012). *C. savignyi Dmrt* mutants present abnormalities in the development of the larval sensory vesicle (Tresser et al., 2010) and the expression of the *C. robusta* ortholog is under early FGF control (Imai et al., 2006). The *KlB* fragment harboring a mutated Dmrt site (*K_Dmrt_mut*) drives GFP expression only in 20% of electroporated larvae, roughly half of the activity of wild-type *KlB* (Figure 4E). Additionally, two binding sites attributed to the homeodomain transcription factors caught our attention (ATTA, blue in Figure 4A). Among TFs able to bind these sites we focused on *Msx* because of its early expression in *C. robusta* pigment cell precursors (Aniello et al., 1999; Russo et al., 2004), and given that it is sharply down-regulated when FGF is blocked (Racioppi et al., 2014). We detected co-expression of *Msx* and *Klhl21/30* in the PCPs at early tailbud stage (Figures 4D–D”’) As development proceeds, the expression of *Msx* in PCP decrease becoming excluded from most of the a9.49 derivatives in 12 hpf tailbud embryos as already described (Racioppi et al., 2014). This early expression suggests that Msx might be activating early *Klhl21/30* transcription, even though Msx factors normally have been described to act as repressors in *Ciona* (Roure and Darras, 2016). We individually mutated each putative Msx binding site in *KlB*, resulting in two mutant constructs, which we termed *K_Msx_mut1* and *K_Msx_mut2*. The mutated reporters caused a decrease in the percentage of GFP-expressing larvae of 24 and 19%, respectively, with respect to the wild-type (Figure 4E). Mutating both putative Msx sites simultaneously caused a strong reduction in GFP expression, observed in only 4% of larvae (Figure 4E, *K_2xMsx_mut*). Taken together, our data suggest that also Dmrt and Msx could be involved in the transcriptional activation of *Klhl21/30* with a novel role in *Ciona*.

### Functional Analysis of *Trans*-Acting Factors by CRISPR/Cas9

To test the potential role of Mitf, Dmrt and Msx as transcriptional activators of *Klhl21/30*, we used CRISPR/Cas9-mediated mutagenesis to knock out these factors in *C. robusta* embryos (Stolfi et al., 2014; Gandhi et al., 2017, 2018). To design primers to generate sgRNAs for *Mitf*, *Dmrt*, and *Msx*, we used CRISPOR v4.0 portal (Haeussler et al., 2016), which takes in account the potential single-nucleotide polymorphisms (SNPs) and the specificity of each sgRNA and provides their predicted “efficacy” (predicted efficiency of their ability to induce specific DSBs) by the Fusi/Doench algorithm (Doench et al., 2016). Using a series of candidate sgRNAs, we targeted different parts of the coding regions of the selected genes (Supplementary Table S3), synthesized *U6* > *sgRNA* cassettes through One-Step Overlap PCR (OSO-PCR) and validated them by peakshift analysis (Gandhi et al., 2017; see section “Materials and Methods” for details; Supplementary Table S3).

**FIGURE 5:**
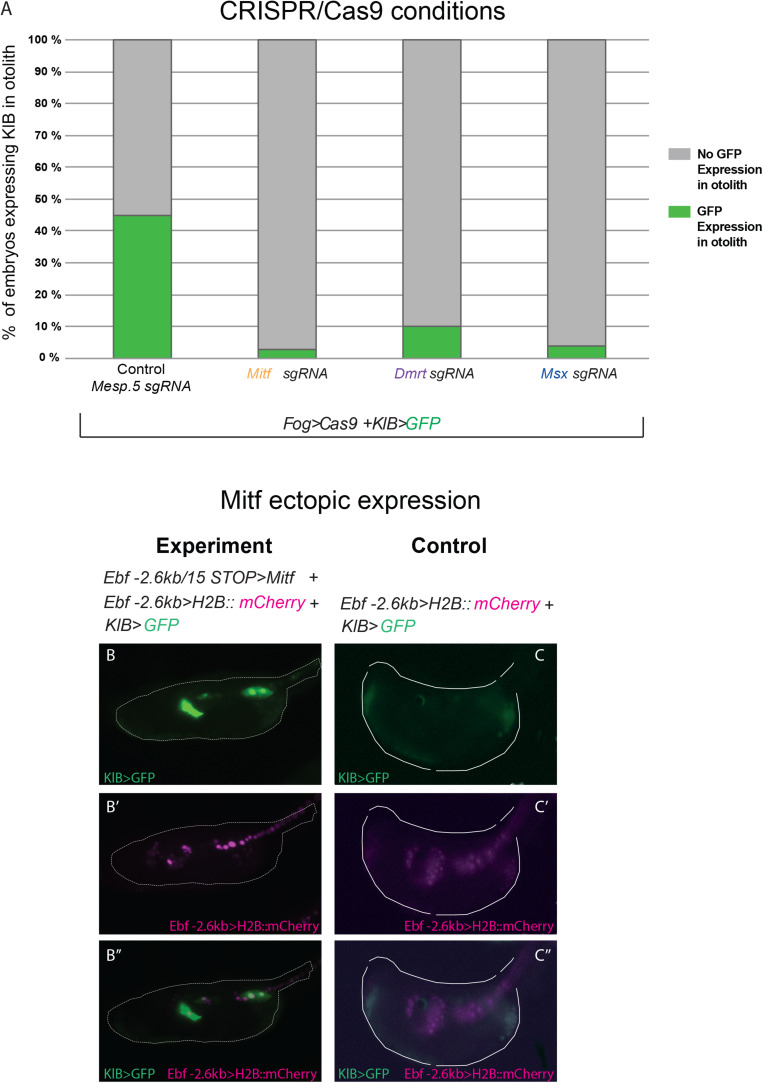
Functional approach on *Klhl21/30* regulators. **(A)** Histogram with phenotypic assays for otolith-specific loss of KlB > GFP in F0 embryos. Larvae were electroporated with 35 μg *Fog* > *Cas9*, 60 μg *KlB* > *GFP* plus 30 μl of OSO-PCR-based sgRNA cassettes *U6* > *Mesp.5* (control), *U6* > *Mitf*-*ex3 106*, *U6* > *Dmrt-ex2 76*, and *U6* > *Msx-ex3 209*, respectively. **(B,C)** Mitf overexpression driving ectopic KlB > GFP expression: control (60 μg *KlB* > *GFP*, 10 μg *Ebf –2.6 kb* > *H2B:mCherry*), overexpression experiment (60 μg *KlB* > *GFP*, 10 μg *Ebf –2.6 kb* > *H2B:mCherry*, 50 μg *Ebf –2.6 kb/* + *15 STOP* > *Mitf*). **(B–B”)** Ectopic expression of KlB > GFP in EBF + cells expressing Mitf ectopically. **(C–C”)** Control larva showing KlB > GFP expression in the otolith **(C,C”)** but no ectopic expression in EBF + cells **(C”)**. Experiments were performed at least three times and 100 embryos were scored each time; each site-directed mutagenesis was significant versus the control *Mesp.5* (Fisher exact test, *p* < 0.00001).

We co-electroporated each gene-specific *U6* > *sgRNA* cassettes, (targeting either *Mitf, Dmrt*, or *Msx*) together with *Fog* > *Cas9*, which drives Cas9 expression in the a-line blastomeres through the *Fog* promoter (Rothbächer et al., 2007), and the *KlB* > *GFP* reporter plasmid to verify the effect of the experiments on *Klhl21/30* isolated regulatory region. As a control, we co-electroporated *Fog* > *Cas9* and *KlB* > *GFP* with an sgRNA cassette targeting the mesoderm-specific transcription factor *Mesp.5* (Davidson et al., 2005), which is not expressed in the pigment cell lineage. While the control sample had a proportion of GFP-expressing larvae comparable to electroporation with *KlB* > *GFP* alone (43% Figure 5A, compared with Figure 2C), we detected a loss of GFP fluorescence in larvae electroporated with *sgRNA* cassettes targeting the three putative regulators of *Klhl21/30* (Figure 5A). When *Mitf* was knocked out, *KlB* > *GFP* was expressed in only 3% of larvae, a sharp decrease compared to the control condition. Moreover, the knockout of *Dmrt* and *Msx* also reduced GFP expression, with only 10 and 4% of larvae showing the *KlB* > *GFP* expression, respectively (Figure 5A). Because of the strong effect of Mitf loss-of-function on *KlB* > *GFP* activity in *C. robusta*, and the evolutionary conservation of *Mitf* function in pigment cell development (Goding, 2000; Levy et al., 2006), we performed a complementary Mitf gain-of-function experiment. We co-electroporated *KlB* > *GFP* along with *Ebf −2.6 kb* > *H2B:mCherry* and *Ebf −2.6 kb/* + *15 STOP* > *Mitf* to overexpress Mitf in *Ebf* + neural progenitors during development. These resulted in ectopic *KlB* > *GFP* expression in *Ebf* + cells in the central nervous system (Figures 5B–B”), suggesting that Mitf drive the transcription of *Klhl21/30* in these cells.

Thus, these data strongly suggest that, downstream the FGF signaling, *Klhl21/30* is under the control of Mitf, with Dmrt and Msx acting as important co-activators.

## Discussion

Our work sheds light on a novel *Ciona robusta* marker of pigment cell development, the gene *Klhl21/30*, which represents the earliest gene specifically restricted to the otolith pigmented cell during development. We show that *Klhl21/30* is a highly conserved member of the poorly studied *Klhl* family, whose components have been implicated in various protein-protein interaction and ubiquitination functions. Although human genome encompasses 42 *Kelch-like* genes (Dhanoa et al., 2013; Figure 1), our phylogenetic analysis revealed the basic chordate toolkit of *Klhl* genes (Figure 1), whose roles, during development, have yet to be fully explored. We described the *Klhl* toolkit of *C. robusta* (32), the larvacean tunicate *O. dioica* (14), and the cephalochordate (amphioxus) *B. belcheri* (37). Thus, we deduced that 25 ancestral subfamilies were present at the stem of chordates and are conserved in human, plus one (*Klhl5L*) tunicate-specific. Despite the tendency of tunicates to lose genes and the relative genomic stasis of cephalochordates (Putnam et al., 2008), we detected several local duplications in these groups. For instance, in tunicates the subfamily *Klhl9/13* has undergone tremendous increase, while in amphioxus we found *Klhl21/30* duplication and a dramatic expansion of *Klhl42* genes. With respect to the ancestral chordate *Klhl* toolkit, larvaceans lost more than 50% of subfamilies, while we registered an expansion of the family in amphioxus, *Ciona* and human. Interestingly, the *Klhl* increase in invertebrate chordates has been driven by different events of gene duplication, whilst WGDs (Abi-Rached et al., 2002; Dehal and Boore, 2005) could have been a major role in modeling human (and vertebrate) *Klhl* repertoire. Further investigations in other species might be important to understand the exact role exerted by gene duplications, WGDs and losses in shaping the *Klhl* toolkit, especially in vertebrates. Taken together, our data represent the most detailed analysis of the evolutionary landscape of *Klhl* family in Chordata so far. Strikingly, *Klhl21/30* is one of the most conserved subfamilies and our syntenic survey demonstrates its conservation in all the tunicates (Supplementary Figure S2). Furthermore, *Klhl21/30* represents one of few subfamilies retained by the gene-loser *O. dioica*, hinting at its relative importance. Moreover, we detected a WGD-origin of human (and vertebrates) *KLHL21* and *KLHL30* genes (Supplementary Figure S3).

There is scarcity of information about the function and expression of genes of the *Klhl21/30* subfamily. Klhl21 is known to bind E3 ubiquitin ligase through Cul3 (Maerki et al., 2009; Courtheoux et al., 2016) and *Klhl30* has been identified as a circadian pathway gene involved in the onset of glioma (Madden et al., 2014). Like many other *Klhl* members, *Klhl21* is implicated in diverse types of carcinoma, probably due to its role in cell division (Shi et al., 2016; Huang et al., 2017; Chen et al., 2018). Our findings represent the first expression data for *Klhl21/30* subfamily in the chordate nervous system. In *C. robusta*, *Klhl21/30* is initially transcribed in two cells of the pigment cell lineage alongside well-known markers of pigmented cells such as *Tyr*, *Tyrp*, and *Rab32/38* (Coppola et al., 2016; Racioppi et al., 2019), which are conserved in chordate pigment cell evolution (Esposito et al., 2012; Coppola et al., 2016). Interestingly, *Klhl21/30* became restricted to the otolith precursor from middle tailbud stage onward. This renders *Klhl21/30* the earliest marker of *Ciona* otolith so far identified, well before the activation of βγ*-crystallin* in the otolith at the larval stage (Shimeld et al., 2005). Expression data from invertebrates and vertebrates could be crucial to understand the potential functional conservation during evolution of poorly studied *Klhl21/30* genes.

The sustained expression of *Klhl21/30* in the otolith prompted us to investigate the transcriptional regulation of this gene. Taking advantage of genome conservation with the sibling species *C. savignyi*, we identified the smallest region containing the TFBS crucial for *Klhl21/30* expression in the otolith. Our *cis-*regulatory mutational analyses suggested the involvement of three transcription factors (Mitf, Dmrt, Msx) in the activation of *Klhl21/30* (Figure 3). The tissue-specific, CRISPR/Cas9-mediated loss-of-function of *Mitf, Dmrt* and *Msx* resulted in highly penetrant downregulation of the *Klhl21/30* reporter in electroporated larvae, suggesting that *Klhl21/30* expression depends on these *trans*-acting factors (Figure 5A). Moreover, a gain-of-function experiment confirmed Mitf as the main regulator for *Klhl21/30* reporter expression in other cells of the nervous system. Due to the fact that some Ebf + cells also express *Msx*, it may be that also Msx collaborates in the ectopic activation of *Klhl21/30* reporter. On the other hand, the low efficiency of ectopic expression of Klhl21/30 in Ebf > Mitf ectopic experiment could reflect lack of the Msx or Dmrt in these cells or low penetrance of CRISPR/Cas9 for these genes. We therefore conclude that Mitf, a key regulator of melanocyte development and melanoma in vertebrates (Levy et al., 2006), seems to be an indispensable transcription factor for *Klhl21/30* expression in *Ciona*, with Dmrt and Msx acting as potential co-factors. However, these transcription factors could also work without a direct interaction with the regulatory region responsible for *Klh21/30* expression in pigment cell lineage.

Interestingly, *Mitf* shows sustained expression in the otolith at later developmental stages (Figure 4; Abitua et al., 2012) confirming that this factor could be the final node of the regulatory network underlying the fate determination between otolith and ocellus pigment cells in *Ciona* as already suggested by Abitua et al. (2012). Besides, Mitf controls also the expression of *Rab32/38*, a conserved melanogenic marker (Racioppi et al., 2019). The role of Mitf in pigment cell development has been observed also in *Drosophila melanogaster* (Hallsson et al., 2004), and the function of Mitf as master regulator of genes related to pigment cells and pigmentation is conserved in vertebrates (Levy et al., 2006; Vachtenheim and Borovanský, 2010). However, in this latter Mitf plays a crucial role also in other processes as mast cell development (Morii, 2004; Putnam et al., 2008) and osteoclast biogenesis (Hershey and Fisher, 2004), while in tunicates this bHLH factor seems to be involved only in pigment cell development, and otolith specification, as suggested also by its specific expression during embryogenesis (Figures 4B–C”’; Yajima et al., 2003). If we consider also the specific expression of *Mitf* in the first pigmented spot of the early-branching amphioxus (Yu et al., 2008), we can speculate that the ancestral *Mitf* function in early development of chordates was related only to pigment cells and, in vertebrates, it acquired new specializations. However, it is not known if tunicates possess cell types homologous to mast cells or osteoclasts, especially in the poorly studied adult phase. Therefore, a more thorough understanding of Mitf functions (and the existence of other PCPs-specific genes regulated by Mitf) throughout the entire life cycle of invertebrate chordates will be necessary to corroborate this hypothesis.

Our data hinted at Dmrt and Msx as positive regulators of *Klhl21/30* (Figures 4, 5). Activated very early by FGF signaling (Imai et al., 2006), the *Dmrt* gene is crucial for otolith development in the sister species *C. savignyi*, in which *Dmrt* mutants typically possess a single ocellus pigment cell but lack an otolith (Tresser et al., 2010). Our results further suggest that Dmrt is a co-activator of an otolith-specific gene, *Klhl21/30.* With respect to Msx, this transcription factor is traditionally considered a repressor in *Ciona* (Roure and Darras, 2016) and in different vertebrates (Takeda et al., 2000; Takahashi et al., 2001; Xie et al., 2013). However, in mouse, Msx1 and Msx2 are the transcriptional activators of the proneural gene *Atoh1* during spinal cord patterning (Duval et al., 2014). Our findings reveal that the function as activator for Msx might be present also in tunicates.

Importantly, while Mitf has clearly a role in *Klhl21/30* activation in *Ciona*, the fragment containing solely Mitf binding sites (*KlE*) is not sufficient to drive expression of *Klhl21/30* in otolith. In contrast, fragments that include Dmrt and Msx binding sites in addition to Mitf binding sites are sufficient for reporter gene activity and one of them (*KlB* > *GFP*) has been utilized for Mitf gain-of-function experiment (Figures 5B–C”). Together with our *cis-*regulatory mutational and CRISPR/Cas9-mediated knockout data, this suggests that all three factors could be key activators. Alternatively, the much earlier expression of *Dmrt* and *Msx* in PCPs evokes that their early binding to the *Klhl21/30 cis-*regulatory region might be required for later transcriptional activation by otolith specifier Mitf, acting as a so-called “pioneer factors” (Zaret and Carroll, 2011), involved in keeping the chromatin accessible to recruit other late transcriptional activators. Novel functional data regarding *Dmrt* and *Msx* in *Ciona* could unveil their function as activators and/or pioneer factors, and if this role is restricted to pigment cells or if it extends to more broadly anterior neural plate derivatives.

In sum, we provided the first description of a *Klhl* member in *Ciona robusta*, identifying it as the earliest otolith pigment cell marker and we elucidated the gene regulatory network (GRN) controlling its expression.

## Materials and Methods

### Evolutionary Analyses and Transcription Factor Binding Site Analysis

Kelch-like protein sequences from vertebrate *Homo sapiens* were used as queries in BLASTp and tBLASTn searches in ANISEED (Brozovic et al., 2018), NCBI or Ensembl genome databases of selected species. Orthology was initially assessed by reciprocal best blast hit (RBBH) approach and supported by phylogenetic analyses. Evolutionary reconstructions were performed using ML inferences calculated with PhyML v3.0 and automatic modality of selection of substitution model (Guindon et al., 2010) using protein alignments generated with MUSCLE (Edgar, 2004) and ClustalX (Larkin et al., 2007) programs. Alignment of Supplementary Figure S1 has been carried out employing ClustalX. Full protein sequences were used in our analysis. Accession numbers and names for phylogenetic tree of Figure 1 are listed in Supplementary Table S1, while those excluded are encompassed in Supplementary Table S2. The analysis of synteny conservation (shown in Supplementary Figures S2, S3) was performed by employing ANISEED, Ensembl and Genomicus databases (Louis et al., 2015). To predict putative transcription factor binding sites (TFBS) in the surveyed cis-regulatory regions, we utilized the MatInspector module of the Genomatix Software Suite and CIS-BP employing a *Ciona intestinalis* (former name for *Ciona robusta*) DNA-binding-domain classes database (Weirauch et al., 2014). We also used the JASPAR database (Khan et al., 2018) to recognize the potential binding sites.

### Animals and Embryo Electroporation

Adults of *Ciona robusta* were collected from the Gulf of Naples, or from San Diego, CA, United States, by M-REP. Gametes from many animals were gathered separately for *in vitro* cross-fertilization followed by dechorionation and electroporation as previously illustrated (Christiaen et al., 2009a; Racioppi et al., 2014). Electroporated plasmid amounts (e.g., 10 μg) were per 700 μl of total volume. Embryos were staged according to the developmental timeline shown in Hotta et al. (2007). To visualize GFP, embryos were fixed in MEM-FA (3.7% methanol-free formaldehyde, 0.1 M MOPS pH 7.4, 0.5 M NaCl, 2 mM MgSO4, 1 mM EGTA) for 30 min and washed several times in PBS-NH_4_Cl and in PBS containing 0.05% Triton X-100. Each electroporation of Figures 3, 4 was carried out using 60 μg of plasmid. The statistical significance of electroporations associated to Figure 4 was validated using Fisher exact test. The statistical significance of electroporations of Supplementary Figure S4 was evaluated employing Chi-square test for trend.

### *In situ* Hybridization

Single and double *in situ* hybridization experiments were performed out essentially as described previously (Christiaen et al., 2009b; Racioppi et al., 2014), using DIG- and FLUO-labeled riboprobes, anti-DIG-POD and anti-FLUO-POD Fab fragments (Roche, Indianapolis, IN), and Tyramide Amplification Signal with Fluorescein (Perkin Elmer, MA). The antisense riboprobes were obtained from plasmids contained in the *C. intestinalis* gene collection release I: *Klhl21/30* (KH2012:KH.L84.23, GC17e22), *Tyrp1/2a* (KH2012:KH.C8.537, GC31h05), *Mitf* (KH2012:KH.C10.106, GC28k08), *Dmrt* (KH2012:KH.S544.3, GC02f18), and *Msx* (KH2012:KH.C2.957, GC42h24) (Satou et al., 2002).

### Molecular Cloning

The *cis*-regulatory elements upstream *Klhl21/30* were PCR-amplified from genomic DNA and their localization on upstream sequence is shown in Supplementary Figure S6. Insertion of the products into pSP72 vector containing GFP (Zeller et al., 2006) was carried out using TOPO-TA Cloning kit (Invitrogen). The QuickChange Site-Directed Mutagenesis Kit (Agilent) was employed to generate the mutations inside the putative binding sites (Mitf, Dmrt, Msx) identified in the sequence of the *KlB* element (Figure 4). All the oligos used for cloning the putative regulatory regions and for mutational experiments are listed in Supplementary Table S4.

### Functional Experiments by CRISPR/Cas9 and Gain-of-Function

*Cas9* and *sgRNA* expression vectors were constructed or used as previously described (Stolfi et al., 2014; Gandhi et al., 2017). The predictive algorithm used for designing *in vivo*-transcribed *sgRNAs* has been the Fusi/Doench (Doench et al., 2016), available on CRISPOR portal (Haeussler et al., 2016). The target sequences have been selected not too close to the translational start, to have the higher impact on the function of protein of interest (Gandhi et al., 2017). DNA oligos used to generate sgRNAs are listed in Supplementary Table S3. One-step overlap PCR (OSO-PCR) was employed for the fast synthesis of a *U6* > *sgRNA* cassettes through a single PCR reaction, performed using Platinum Pfx Polymerase (Invitrogen). The products were cloned into pCESA plasmid using Gibson Assembly Cloning Kit (NEB). Before the electroporation, the products were purified utilizing AMPure XP (Agencourt).

*Mitf*, *Msx*, and *Dmrt* sgRNAs were validated by PCR amplification and Sanger sequencing of the targeted region as previously described (Gandhi et al., 2018). Pooled larvae were lysed for 30 min in 180 μl buffer + 5 μl of Proteinase K from QIAamp DNA Micro Kit (Qiagen) and eluted in 20 μl of water. Approximately 200 ng/μl of genomic DNA extracted from hatched larvae was used for “touchdown” PCRs with Platinum Pfx Polymerase (Invitrogen), as described in Gandhi et al. (2017). The genomic oligos (“peakshift oligos”) employed for “touchdown” PCRs were selected at 150–500 bp away from the primer (Supplementary Table S3), to ensure a proper peakshift. Electroporations were performed as single biological replicates. Electroporation mix recipes can be found in the Supplementary Figure S7. The statistical significance of electroporations associated to Figure 5 was validated using Fisher exact test. Mitf overexpression construct (*Ebf-2.6kb/* + *15 STOP* > *Mitf*) was designed as described in Supplementary Figure S7: STOP indicates the presence of stop codon between the ATG of *Ebf* and the *Not*I site where *Mitf* was cloned (no amino acid sequence of Ebf is included in Mitf protein). Images were captured using Confocal ZEISS LSM 700 or ZEISS Apotome.2 compound microscopes.

## Data Availability Statement

All datasets presented in this study are included in the article/Supplementary Material.

## Author Contributions

UC and FR: conceptualization. UC and AK: investigation. UC, AS, and FR: data curation. AS and FR: supervision. UC: writing. AS and FR: review and editing. All authors contributed to the article and approved the submitted version.

## Conflict of Interest

The authors declare that the research was conducted in the absence of any commercial or financial relationships that could be construed as a potential conflict of interest.

## Supplementary Material

FIGURE S1 Domain conservation among Klhl21/30 proteins of *Ciona robusta* and *Homo sapiens*. BTB/POZ (bold), BACK (underlined), Kelch-repeat 1 (green), Kelch-repeat 2 (yellow), Kelch-repeat 3 (turquoise), Kelch-repeat 4 (magenta), Kelch-repeat 5 (gray). Domains mapped using Smart classification available on Ensembl database (*H. sapiens* KLHL30: ENSP00000386389.1; *H. sapiens* KLHL21: ENSP00000366886.4; *C. robusta* Klhl21/30: ENSCINP00000014893.3). *Ciona* Klhl21/30 shares the same domain architecture of human KLHL (according to Dhanoa et al., 2013).

FIGURE S2 *Klhl21/30* synteny analysis in Olfactores. Identification of an ancestral cluster in ascidians, comprising *Klhl21/30* gene (orange boxes). Ascidians and human share the gene duplet formed by *Klhl21/30* and *Nphp4* (red rectangle). Each orthologous gene has been represented using the same color code and black arrows for transcription direction.

FIGURE S3 *Klhl21/30* synteny analysis in Gnathostomes. Conservation of genes flanking gnathostome *Klhl21* and *Klhl30* (orange boxes) comparing *Latimeria chalumnae*, *Lepisosteus oculatus*, *Xenopus tropicalis* and *Homo sapiens*. Each orthologous gene has been represented using the same color code and black arrows for transcription direction.

FIGURE S4 Expression of *Klhl21/30* in embryos electroporated with *dnFGFR*. Evaluation of percentage of embryos showing expression in the otolith respect to wild-type (wt). The experiment was repeated three times, showing a significant trend respect to control (Chi-square test for trend, *p* < 0.0001; *n* = 200).

FIGURE S5 *GFP* transcription in tailbud embryos electroporated with *KlA >GFP*. *In situ* hybridization experiment using *GFP* probe on tailbud embryos electroporated with *KlA >GFP* construct **(A,B)**.

FIGURE S6 Localization of regions used for *cis*-regulatory analysis (Figure 3) inside upstream region of *Ciona robusta Klhl21/30* (KH.L84.23). Different colors indicate the selected regions and the oligos used for cloning, with respective combinations.

FIGURE S7 Electroporation mixes and constructs used for functional experiments.

TABLE S1 List of Klhl protein sequences employed for phylogeny of Figure 1.

TABLE S2 Klhl protein sequences excluded from phylogeny for their high degree of molecular divergence. Their names were assessed by genome search and reciprocal BLASTs.

TABLE S3 **(A)** List of oligonucleotides used for functional assays. Names contain indication on the position of the target sequence inside the selected exon (e.g., Mitf-ex3 106 indicates that the target sequence is within exon3 starting from its nucleotide 106). In bold and capital letters the sgRNA N (19) target sequences. In lowercase the protospacer sequence (for OSO PCR) appended 3′ to a forward primer and, in reverse complement, appended 3′ to the reverse primer (Gandhi et al., 2017). In red are highlighted the oligos whose sgRNAs were able to cause deletions. “Specificity” indicates the capability of the synthetized sgRNA to target a specific sequence (0–100); “efficacy” represents a prediction of sgRNA ability to induce Cas9-mediated DSBs (0–100); “SNPs” is the number of potential single-nucleotide polimorphisms associated to each sgRNA, which can possibly affect its pairing to the target. All these values were retrieved from CRISPOR portal. **(B)** List of “peakshift” genomic oligos (in blue) used to verify the effect of deletions on specific exons (ex i.e., exon).

TABLE S4 List of oligos used for cloning and mutational experiments (in red are shown the mutated nucleotides, with respect to wt sequence).
