# Supplementary material for: The Cis-Regulatory Code for Kelch-like 21/30 Specific Expression in Ciona robusta Sensory Organs
Source: Front Cell Dev Biol. 2020 Sep 11;8:569601. doi: 10.3389/fcell.2020.569601 (PMC7517041; doi:10.3389/fcell.2020.569601)
Supplement: FIGURE S1 — Domain conservation among Klhl21/30 proteins of Ciona robusta and Homo sapiens. BTB/POZ (bold), BACK (underlined), Kelch-repeat 1 (green), Kelch-repeat 2 (yellow), Kelch-repeat 3 (turquoise), Kelch-repeat 4 (magenta), Kelch-repeat 5 (gray). Domains mapped using Smart classification available on Ensembl database (H. sapiens KLHL30: ENSP00000386389.1; H. sapiens KLHL21: ENSP00000366886.4; C. robusta Klhl21/30: ENSCINP00000014893.3). Ciona Klhl21/30 shares the same domain architecture of human KLHL (according to Dhanoa et al., 2013). [file Image_1.PDF]

Figure S1. Domain conservation among Klhl21/30 proteins of *Ciona robusta* and *Homo sapiens*. BTB/POZ (bold), BACK (underlined), Kelch-repeat 1 (green), Kelch-repeat 2 (yellow), Kelch-repeat 3 (turquoise), Kelch-repeat 4 (magenta), Kelch-repeat 5 (grey). Domains mapped using Smart classification available on Ensembl database (*H. sapiens* KLHL30: ENSP00000386389.1; *H. sapiens* KLHL21: ENSP00000366886.4; *C. robusta* Klhl21/30: ENSCINP00000014893.3). *Ciona* Klhl21/30 shares the same domain architecture of human Klhl21 (according to Dhanoa *et al.*, 2014).

```
KLHL30    Homo sapiens           -------------------------------MVRNVDDLDFHLPSHAQDMLDGLQRLRSQ    29
KLHL21    Homo sapiens           -----------------------------MERPAPLAVLPFSDPAHALSLLRGLSQLRAE    31
Klhl21/30 Ciona robusta          LVAIVGYSLNWLESERLKASPSKSVCMMLDIKTTTAHSSTFVKPNFSNEIVQGLRNLRTD    60
                                                                         *  *  .:  .:: ** .**::

KLHL30    Homo sapiens           PKLADVTLLVG-GRELPCHRGLLALSSPYFHAMFAGDFAESFSARVELRDVEPAVVGQLV    88
KLHL21    Homo sapiens           RKFLDVTLEAAGGRDFPAHRAVLAAASPYFRAMFAGQLRESRAERVRLHGVPPDMLQLLL    91
Klhl21/30 Ciona robusta          GKFFDIVIHVA-DERFPCHRIILAAASNYFRSMFAGMMKESCTDKVTLFGVEPEAAEILI    119
                                  *: *:.: .. .. :*.** :** :* **::**** : ** : :* * .* *     *:

KLHL30    Homo sapiens           DFVYTGRLTITQGNVEALTRTAARLHFPSVQKVCGRYLQQQLDAANCLGICEFGEQQGLL    148
KLHL21    Homo sapiens           DFSYTGRVAVSGDNAEPLLRAADLLQFPAVKEACGAFLQQQLDLANCLDMQDFAEAFSCS    151
Klhl21/30 Ciona robusta          EFCYTGTAVITENNVIELLKASDLLQFHSVREACCEFVGKRLDSSNCLSVRQFAEKLSCA    179
                                 :* ***   .:: .*.   * :::   *:* :*::.*   :: ::** :***.: :*.*   .

KLHL30    Homo sapiens           GVAAKAWAFLRENFEAVAREDEFLQLPRERLVTCLAGDLLQVQPEQSRLEALMRWVRHDP    208
KLHL21    Homo sapiens           GLASAAQRFILRHVGELGA-EQLERLPLARLLRYLRDDGLCVPKEEAAYQLALRWVRADP    210
Klhl21/30 Ciona robusta          KLADEALTYTAENIVTVACSQEFNSLNYQQIRPILAHHMLNIDKEEGAFEILMQWLKCDI    239
                                  :*  *  :  .:.  :.  :::  *    ::    *  . * :  *:.  :  ::*:: *

KLHL30    Homo sapiens           QARAAHLPELLSLVHLDAVPRPCVQQLLASEPLIQESEACRAALSQGHDG----------    258
KLHL21    Homo sapiens           PRRAAHWPQLLEAVRLPFVRRFYLLAHVEAEPLVARCPPCLRLLREARDFQAARYDRHDR    270
Klhl21/30 Ciona robusta          ELRKDDFCRLMEVIRLPFIRRIYLLSKIETNPIVRASTLCKKLIKETRLFHTCMLDKNEK    299
                                   *  .  .*:. ::*  : *  :    : ::*::  .  *   : : :

KLHL30    Homo sapiens           -APLAL----QQKLEEVLVVVGGQALEEEEAGEEPTPGLGNFAFYNSKAKRWMALPDFPD    313
KLHL21    Homo sapiens           GPCPRMRPRPSTGLAEILVLVGGCDQDCD--------ELVTVDCYNPQTGQWRYLAEFPD    322
Klhl21/30 Ciona robusta          -TSIRLQPRPSTGIAELLVTVGGCDENCD--------EATTVVSYNPVSGTWKALAQVED    350
                                      :    .  : *:** ***   : :            ..  **  :  *  * :. *

KLHL30    Homo sapiens           YHKWGFSLAALNNNIYVTGGSRGTKTDTWSTTQAWCFPLKEASWKPVAPMLKPRTNHASA    373
KLHL21    Homo sapiens           HLGGGYSIVALGNDIYVTGGSDGSRL----YDCVWRYNSSVNEWAEVAPMLKAREYHSSS    378
Klhl21/30 Ciona robusta          NLRGGYATVSIGNDIYVTEGKKNT--------KVWRYKSQVNEWIKVCNMNYAHEYHGSV    402
                                     *:: .::.*:**** *. .:          .* :  .  .*  *. *   :  *.*

KLHL30    Homo sapiens           ALNGEIYVIGGTTLDVVEVESYDPYTDSWTPVSPALKYVSNFSAAGCRGRLYLVGSSACK    433
KLHL21    Homo sapiens           VLDGLLYVVAADS-----TERYDHTTDSWEALQPMTYPMDNCSTTACRGRLYAIGSLAGK    433
Klhl21/30 Ciona robusta          VLQSYIYVIGSEG-----CEKYDFISDSWTLIAKVPHRVNNCSVAACAGKIYTIGSLYGS    457
                                 .*:. :**:..        * **  :***  :      :.* *.:.* *::* :**    .

KLHL30    Homo sapiens           YNALALQCYNPVTDAWSVIASPFLPKYLSSPRCAALHGELYLIGDNTKKVYVYDPGANLW    493
KLHL21    Homo sapiens           -ETMVMQCYDPDTDLWSLVDCGQLPPWSFAPKTATLNGLMYFVRDDSAEVDVYNPTRNEW    492
Klhl21/30 Ciona robusta          -EEILLQVYSPDRDKWDNIPI-NLPPMSFVPTISSLKGLLYFTSEDSKEVLTFDPRRNEW    515
                                  : : :* *.*  * *. :    **     *  ::*:* :*:  ::: :* .::*  * *

KLHL30    Homo sapiens           QKV-QSQHSLHENGALVPLGDALYVTGGRWQGMEGDYHVEMEAYDTVRDTWTRHGALPRL    552
KLHL21    Homo sapiens           DKI-PSMNQVHVGGSLAVLGGKLYVSGGYDNTFE--LSDVVEAYDPETRAWSVVGRLPEP    549
Klhl21/30 Ciona robusta          INTTSPMLHVHLGGSTTVFGGKLIVSGGYDERYD--LSGIIEAYDTDTNTWSVIGKMPQP    573
                                  :       :* .*: . :*. * *:**  :  :      :****    :*:  * :*.

KLHL30    Homo sapiens           WLYHGASTVFLDVSKWTQPSGPTQEH----------------------       578
KLHL21    Homo sapiens           TFWHGSVSIFRQFMPQTFSGGRGFELDSGSDDMDPGRPRPPRDPDELH       597
Klhl21/30 Ciona robusta          IFWHGCVSIYSFT-SKCFQH----------------------------       592
                                  ::**. :::
```
