# Supplementary figures and images for: The Cis-Regulatory Code for Kelch-like 21/30 Specific Expression in Ciona robusta Sensory Organs
Source: Front Cell Dev Biol. 2020 Sep 11;8:569601. doi: 10.3389/fcell.2020.569601 (PMC7517041; doi:10.3389/fcell.2020.569601)

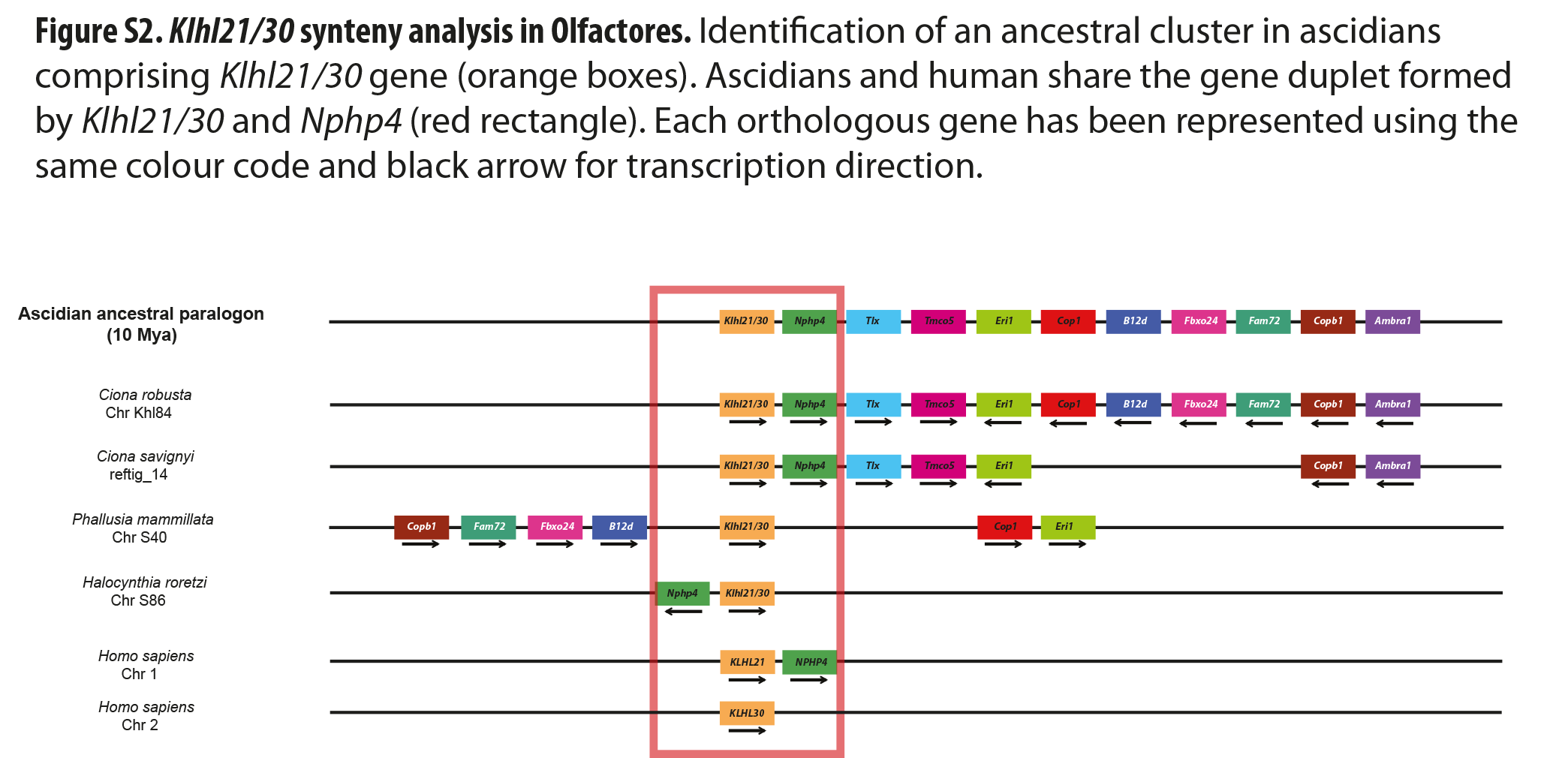

Supplement: FIGURE S2 — Klhl21/30 synteny analysis in Olfactores. Identification of an ancestral cluster in ascidians, comprising Klhl21/30 gene (orange boxes). Ascidians and human share the gene duplet formed by Klhl21/30 and Nphp4 (red rectangle). Each orthologous gene has been represented using the same color code and black arrows for transcription direction. [file Image_2.TIFF]

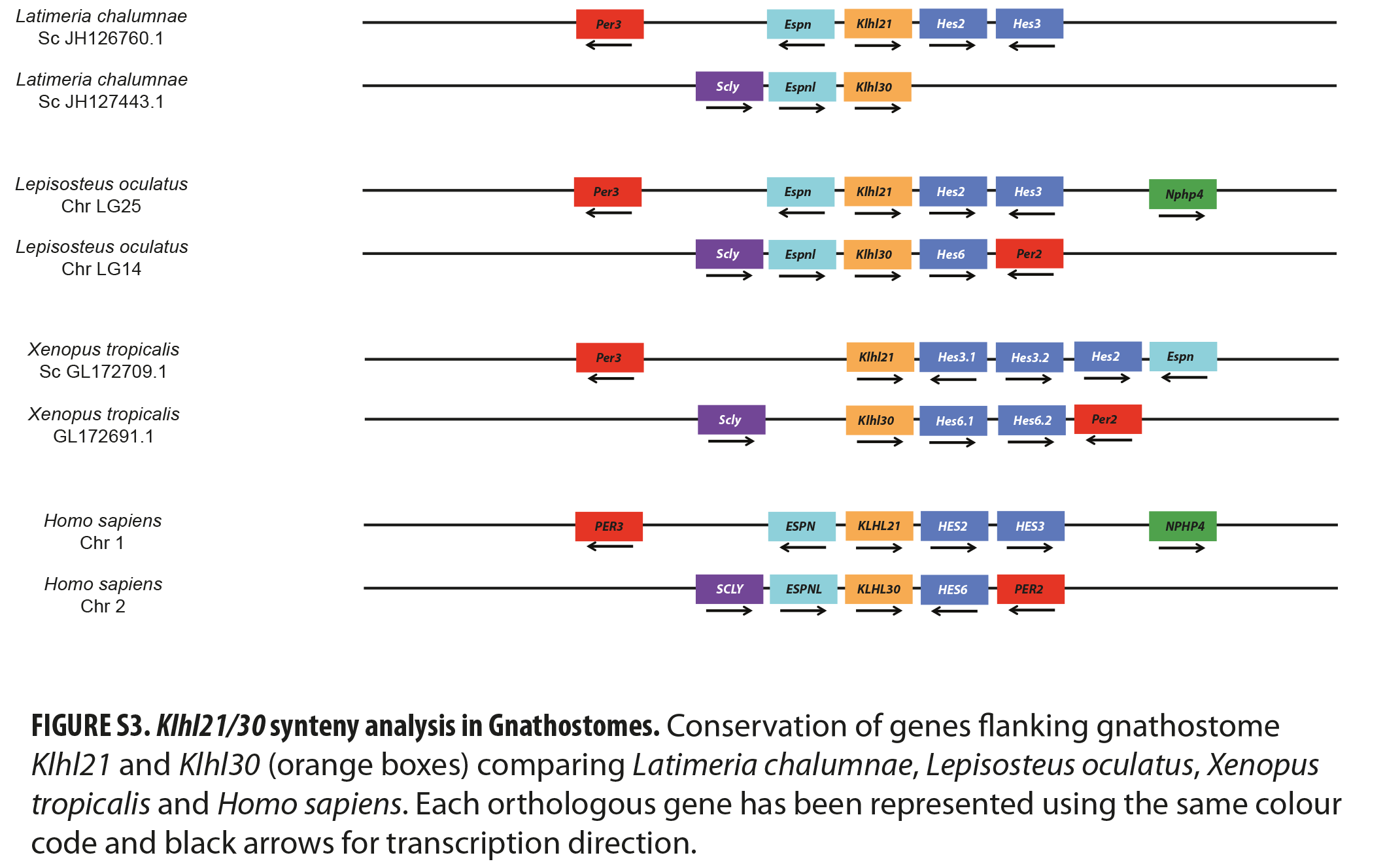

Supplement: FIGURE S3 — Klhl21/30 synteny analysis in Gnathostomes. Conservation of genes flanking gnathostome Klhl21 and Klhl30 (orange boxes) comparing Latimeria chalumnae, Lepisosteus oculatus, Xenopus tropicalis and Homo sapiens. Each orthologous gene has been represented using the same color code and black arrows for transcription direction. [file Image_3.TIFF]

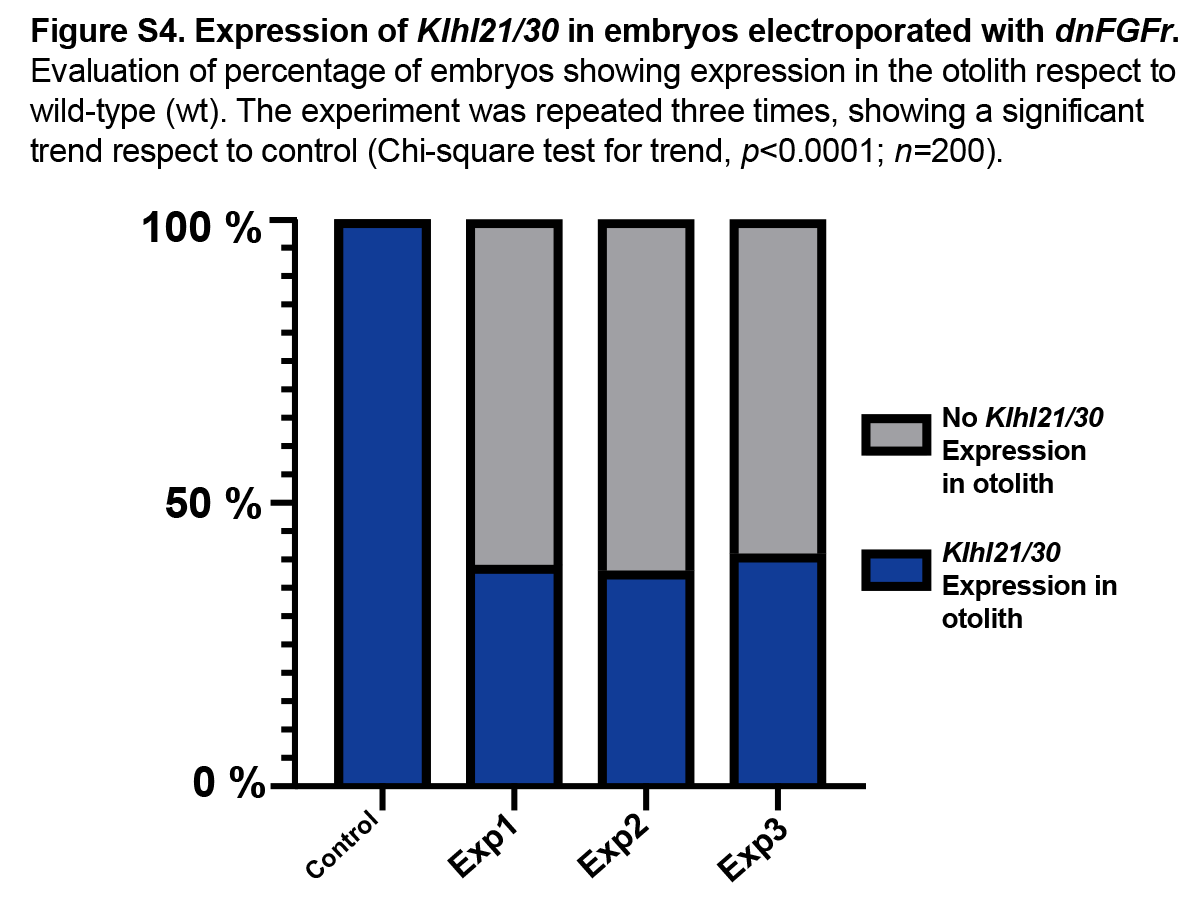

Supplement: FIGURE S4 — Expression of Klhl21/30 in embryos electroporated with dnFGFR. Evaluation of percentage of embryos showing expression in the otolith respect to wild-type (wt). The experiment was repeated three times, showing a significant trend respect to control (Chi-square test for trend, p < 0.0001; n = 200). [file Image_4.TIFF]

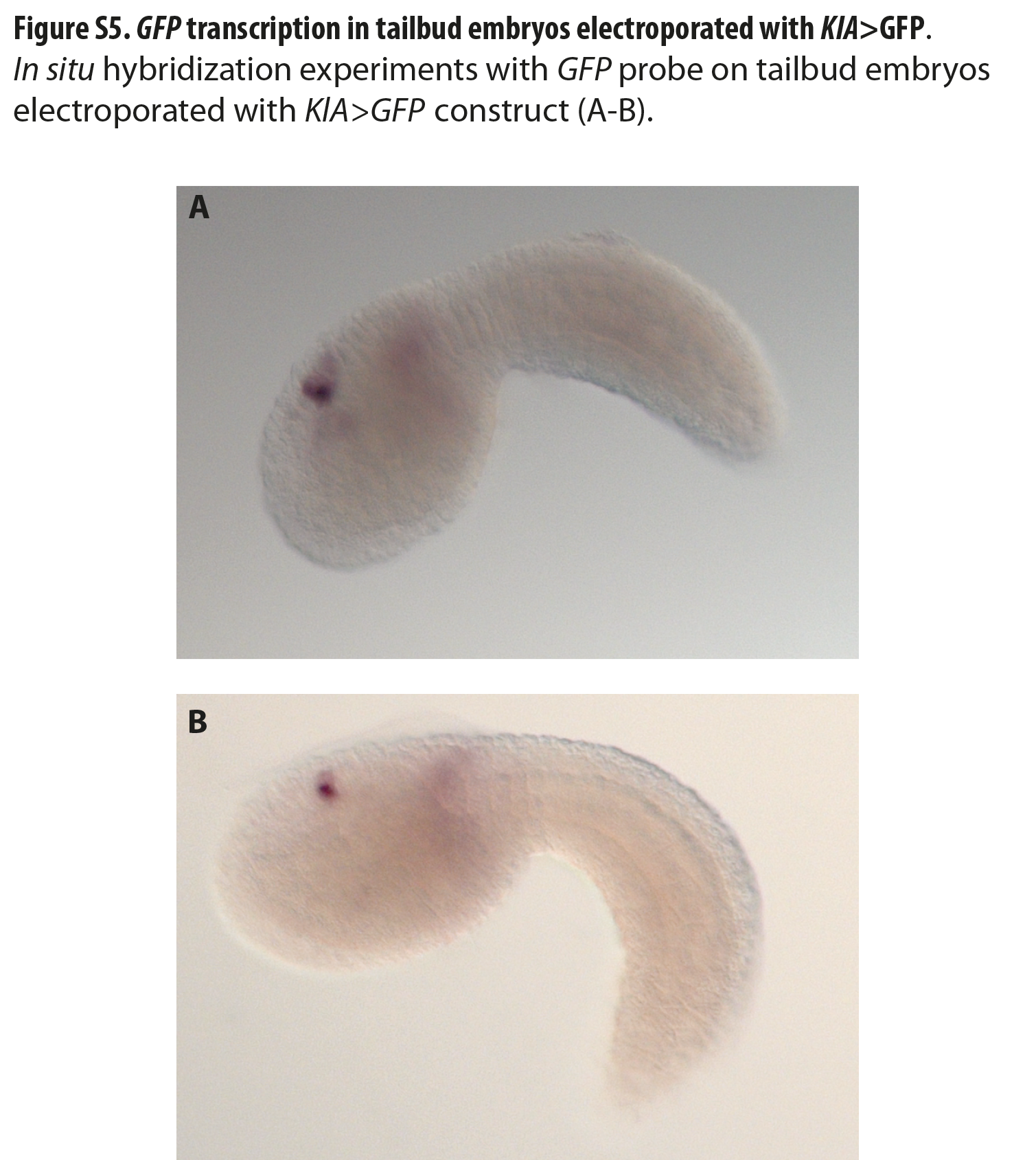

Supplement: FIGURE S5 — GFP transcription in tailbud embryos electroporated with KlA >GFP. In situ hybridization experiment using GFP probe on tailbud embryos electroporated with KlA >GFP construct (A,B). [file Image_5.TIFF]
