# Supplementary material for: The Cis-Regulatory Code for Kelch-like 21/30 Specific Expression in Ciona robusta Sensory Organs
Source: Front Cell Dev Biol. 2020 Sep 11;8:569601. doi: 10.3389/fcell.2020.569601 (PMC7517041; doi:10.3389/fcell.2020.569601)
Supplement: FIGURE S6 — Localization of regions used for cis-regulatory analysis (Figure 3) inside upstream region of Ciona robusta Klhl21/30 (KH.L84.23). Different colors indicate the selected regions and the oligos used for cloning, with respective combinations. [file Image_6.PDF]

Figure S6. Localization of regions used for *cis*-regulatory analysis (Figure 3) inside upstream region of *Ciona robusta Klhl21/30* (KH.L84.23). Different colors indicate the selected regions and the oligos used for cloning, with respective combinations.

>
CGATTATATTTTGCCGTTTCGGAAATCCCTATGTGCTGACACAGCAAAGAACAATTGCTGCCTAAGGTGATCTCACAACG
TCTTATTACGTAATTACATGTTTGCTGAATCTATTCGTCGAGCCCGTTCTCAATAGATTCCAAATTTGGCCGTGACAGTC
GTGATCTAGTCTTGTTTTTGTAGTTT**GCGCACTTATAACATGCA**ATTATATTCTAACGTTTCTTCTTTCT**GTGATCAGTT**
CGACTATACTTTTACATTTATTTCTCAGTCCGTATAATACGAAGTGTACAGTGCCTAATTTA**GTGAGTTAACATTTGTGC**
GTTTGGTCTGTTGCGAATTAACTGATTGTCAAGTTCTAAATCGTTTCGCTTTCTTTCTGCCCTGG**TGCCTAGTCTTGTCA**
CGTGAAAAACACTCAGGGATTATAAATAACCACCCACGATTACTTACGTAATAGCTGAAAATGATTCCCACGTGATGCGA
AGTCATTAAACAAAGAAACAACGTCACGCAAGAACTTTACATGACTAGCTGAAGCAAGCGATGGATTTGTA**TGAGGTTAC**
TAGAACGTTATTATTATACAATTTATGGCACATAGCCTACATGCCAACGATTAAAAGGGATTGTTGAGTATATTTGTTAA
ATAGGCAAACTATATAGATTATGTGCCATCGTCGATTTAAGAATATTTTCCAATGTGTTGGTGCAGGTTCCGCAACGGTT
CGTACGACTGCAAAGTTTTTTTGTTTTTTTTTCTGGGGGTTCGAAGTGATTTACCTTGTTATGACGTCATATGACCAAAA
GTCCCTAAATAGCGATAGTTAGCATAGAAGTCGTGTATAGTGTATAGTGCATTCTGTACAACAGTACGTTTACTTTCGCC
ACATTGACAGGCAATGAGTTCTAGTTGCTATTGTTGGTTATAGTTTAAATTGGTTAGAATCAGAACGCTTGAAAGTTGAG
ATATGTTTATTTGTATCTTTTATAAATGG**CGGTAATGGTATACTTAACC**ATAAATTGTTAGTTGTAACTATATTATATTT
TTTACAGCACAATCAGCTTTCTGAATGCTTGAAGTATATAGCTAGTCCAAGCAAATCTGTTTGC**ATG**ATGCTAGACATCA
AAACAACAACGGCTCATAGTAGCACGTTCGTTAAACCTAATTTTTCGAATGAAATCGTGCAAGGTCTAAGAAACCTTCGA
ACCGATGGTAAATTTTTTGACATTGTTATTCATGTTGCTGACGAACGATTCCCATGCCATAGGATTATCCTGGCAGCAGC

ATG

KLA
CCTAAGGTGATCTCACAACG
GGTTAAGTATACCATTACCG

KLB
GCGCACTTATAACATGCA
TGAGGTTACTAGAACGT

KLC
TATTATTATACAATTTATGG
GGTTAAGTATACCATTACCG

KLD
GCGCACTTATAACATGCA
TCACGTGACAAGACTAGGCA

KLE
TGCCTAGTCTTGTCACGTGA
TGAGGTTACTAGAACGT

KLF
GTGAGTTAACATTTGTGCG
TGAGGTTACTAGAACGT

KLG
GTGATCAGTTCGACTATACT
TGAGGTTACTAGAACGT
