# Supplementary material for: The Cis-Regulatory Code for Kelch-like 21/30 Specific Expression in Ciona robusta Sensory Organs
Source: Front Cell Dev Biol. 2020 Sep 11;8:569601. doi: 10.3389/fcell.2020.569601 (PMC7517041; doi:10.3389/fcell.2020.569601)
Supplement: FIGURE S7 — Electroporation mixes and constructs used for functional experiments. [file Image_7.PDF]

Figure S7. Electroporation mixes and constructs used for functional experiments.

Electroporation mixes:

Mitf **CRISPR/Cas9 KO:**
35 µg *Fog>Cas9*
30 µl of OSO-PCR-based *U6>Mitf-ex3 106* sgRNA cassettes or *U6>Mesp.5* (control) sgRNA
60 µg *KlB>GFP*

Dmrt **CRISPR/Cas9 KO:**
35 µg *Fog>Cas9*
30 µl of OSO-PCR-based, *U6> Dmrt-ex2 76* sgRNA cassettes or *U6>Mesp.5* (control) sgRNA
60 µg *KlB>GFP*

Msx **CRISPR/Cas9 KO:**
35 µg Fog>Cas9
30 µl of OSO-PCR-based *U6>Msx ex3 209* sgRNA cassettes or *U6>Mesp.5* (control) sgRNA
60 µg *KlB>GFP*

Mitf overexpression:
50 µg *Ebf -2.6kb/+15 STOP>Mitf* (or *Ebf>lacZ*[R1] control)
10 µg *Ebf -2.6kb>H2B::mCherry*
60 µg *KlB>GFP*

File containing information (with color code) on cloning of construct *Ebf -2.6kb/+15 STOP>Mitf*.

AscI
Ebf -2619 to +15
Ebf endogenous START codon (ATG)
Inserted STOP codon (TAA)
NotI
Mitf coding sequence
EcoRI

ggcgcgccattcttccgggaaataagagcggcagcgactttattcgaatttcaaatttccatgatattcg
ataccccacactccaccctataacggtgacctggtttaggaaatatgtgacgttgcttcacgacaatttt
agcagctatataagttaccattgcaaaaaagatcagtctttttatttatcacaatttttaccagattttt
aaatattttttagaaataacttaaattttgatcaaactggattaaaattgcaaattttacatttttgtga
taaatattaatctttttagtcgccatggcttaaaaatgtcatttgtttagtccgctgtattcaattttac

cgtttgataaatcaaacggttaacttgcgcataaaaccaatttctcatatggatattactccgcgcgagt
tctaacaacgcgttctcttcgctggccaagatcgatactccgtgacgtcacaatcacatgcgcgcgtctg
aaatatggtcgacagagttgcaacacgcaaccctttttgtttgggttaatttccctctttgtttttttaagt
ccgatcgcgtcgccacatctgttcgacgaatcttcgttctgtcttagcaactttgcggctctgtccattg
tgaagtcgtaaacgaggcattgtcgtcactgctgtctcgcctacgtcacaaagcagtgcacagtgacgtc
acagagacgaggtcgcgtgccttcgagttggaaaattcaaagcattgcacttttgccgaatgttaattt
ttacgacggaagaagttaggacaagcataaaataacaaataaataacccataaaaaacgtaaaaacaaac
aagagaacggtattcaaaattagaatttcgaaaaaaatgttaaaaaataatacttagagtcgctgaacaa
tttaagccgacaaaggccacaaaactgcctaaaatttataaaaaaaatgtaaaattattttgttttttttt
aaactacagttatcacctttaaaacaaacaaattagcaaacgttgtaattacttcacaactttcttgcga
cgctaaaaggcggcgaattttattgctattgtgacgtcacaagcgctctcgtcacgcccggatacgatta
gaacaacgaaggattgtttgtttttaattatttctctgtttaatcatttgatttagcgcggcacaaattt
tgttttatataaaatgtatccatttatctctgcgcgtttttgtactatttttttgaaaaatgtttgttaa
cctttttgaaaatcgcgaaaccaacgaaattatttccaaaggctgtacaattctttttcgttaggttacgt
gtttaagtataggcccaagttttaaatggcggacagagtttcgtattttgatatttgaattttttttgaaa
tcttgaaaaaaaataattgtacgttttacatagaataactaaccaaaatatctgaagcaaaaattacgta
caattttttaaaatgtaatttactttctagcttttaattttttgtgcttttctcaatatgtgtgccatatt
ttaaaaacgtaaatttgcctgttgttaagcggaggaaaaagtaatctgcgtgaatgcgaaaaacacgatt
ttggaatcagcgccggcaatggtgtttgtaaatagggggtggcatacgcgtttcgtagcgaaagagagaat
gaggcgaaagtcgacagatgcacgctccgatttatgagacaggaaccagtcgcgagggcacggaggaaaa
agaaccttactccaagacatgcgccgcctttttttctttctgtcctagtcaggaatactagagtatagaag
gccacgcgtcgtggagtttaaaaccagcaagccagtgtctcaacggacacatcaatacagagacttctct
cagtggacaactcggacgattcgccactaacttggtggattcgtcccggacgacccatgggccgggtccc
agcgcgctagttggccaccatacagtgtagaatcagctagatcgtctctgcggatttcgcaaatagattg
agttggagatagcttcccgaccgggatttcgactaatttgcaatgttagttattaatcaaggtgacagtc
aggagttaaagttaatttacctttttgaaagggcaaaaagattttcgaagtaaattgattccgttaattgt
aactcttaaacgcaaaccgataaacgacgccattttgcttttcattgagaaactaaccatttaggcattc
tataattaaaattaaatgtttttaaaatctgtaactatctgtaataaactagaattatttatttcagttt
aaattttatttaaaacaataatttttattatttcctttattcatcctaatgatattggtatagagaaaac
gatgtttttatttccataaaatttttatttcagaaaagtcattttgttcaatttaaaaaaattcctttattc
taaaaaaaatgcccaaaacaagttctttatttcttaaaaattatcctaaataaaaaaatccacgttttaat
aaaatgtataaaattgaaaactataaaaagatgacttatttttttaccctaacgtgattttcaccagat
accttaagtgttattttatttgtaagtaatatccaaatggcaacaatcgcgtaagcggccgcaaccATGG
GTAGTCGCGCTAACATGAAGATGCAGTTACAAAGGGACAAGGCCCTTATGGAAGAGAAGAGATTAAGACA
AGCTTCACAAAATGCAGCTCAAGCCTCAAAAAGTTCTTCATCTTCAACTATTAACATGCCAGTTGTAACT
CTAACTTCTCCtCCCCAACTACCTGTACAAGTGCTTAAGGTTGAGACTGGCCTACAGAACCCGACCCAAT
ACCATGTTCGTGAAACACAACGTAACCAAGTTCGTGAGTACATCAGTCAGTCTCATAAAGATGGTTCCAA
ATACATGGTCCGCCAGCTgTTCAACCCTTCGCAAAGCGCTCCCACCAACGCAGGTGACGCATTCCATGCT
AACAGTACACCAGCGAGTCCTCTAGCTCGTCTCAACTTGTCCACAGACCCGATGACCGACATtATAGACG
ACATTGTCAGCCTTGAGTCAAGCTTCGGAGACGAGCCTCGCTTCGACCGTAACGCTACAACTCTCCAAAC
AGTTTCCCCGTCTGACCTTGGAAGTTTCGCCCGCTCCTCTGCCCATTCCCTGCCCGTTATCTCCAACGAA
ACGTCCTCATCGTGTCCTGTCATCAAACGTGAGTTTACGGAAGAAGACGCTCGTCTCTTCGCTAAAGATC
GGATCAAGAAGGACAATCACAATATAATTGAGCGGAGACGAAGGTACAATATAAACGATCGAATACGTGA
GTTGGGACACCTCGTGCCAAAGTCGTCTGACCCAGAATTGCGTTGGAATAAAGGTTCCATACTGAAAGCT
GCAGTAGATTACATACAGCACTTACAAAACGACCAACAGAAACATCGTGCACTTGAACAACGAAGTAAAC
AAATGGAGACGATGAATAAGAAGTTACTGTTACGAGTTCAGGAACTCGAAATGACGGTACAACAGTgCGG
TATTAATGTTGACAACAGCGAAAAACAAGGCATGATGAACCAGCTTCTTAACTTTAACACCGCCCCGACC
GCGGGCATGACGGACGGGATGTTCACCGCAGAAGAGGAACACCACGTCGCAAATTTCCCCACCAACGCCG

TCTCGCCCCAGAACAGGTCGCCGGGTACATTAACATCACAAACAGCGGAAATAATAGTTTACCAACAGCC
AGAGCAGGTAGCACCTGGTGCGAATACGAACCCCGACCAGCAGTTTTTACACcaaCAACAACAACAACAA
CAACAACAGTTATTGACGGTCGACTCCACAATCATCAACGACCAACAACAGCCAAGTCTCTCCCCTAACC
ATCAGATGCAGGGTAACTTCTCCCCTGtTAACTATAACCAGCAATCACCTTTGGGCAGTGCAGCTTCTAG
CACTCTCTCGCCAACACACAGTGAAATGAACATGCAACAACAACAACAACAGCAAcaacaacagCAACAa
CAACAAACACAACAGCAACAACAAGAACAGTCCAACACGTTAAATTTAAATCTcGACGAAGTAGATATGA
CAGCGTTTCAATTTCCCGCAAATAACGATGCCGATCCACTAACCAGTATAATGGGGGCGGGTTCAGCTC
CTTGCTGACTTCAGAGCCAAATCAGTTTAATGACGTCATGATGGTGGACGATTACGGTCTTATAAAAGAC
GATATATTTCTGTCCGATGCTAACCAGTGAgaattccagctgagcgccggtcgctaccattaccagttgg
tctggtgtcaaaaataataataaccgggcaggccatgtctgcccgtatttcgcgtaaggaaatccattat
gtactatttaaaaaacacaaactttggatgttcggtttattctttttcttttactttttatcatggga
gcctacttcccgtttttcccgatttggctacatgacatcaaccatatcagcaaaagtgatacgggtatta
tttttgccgctatttctctgttctcgctattattccaaccgctgtttggtctgctttctgacaaactcgg
aacttgtttattgcagcttataatggttacaaataaagcaatagcatcacaaatttcacaaataaagcat
ttttttcactgcattctagttgtggtttgtccaaactcatcaatgtatcttatcatgtctggatcgacaa
agtcaaagcggccatcagatctgccggtctccctatagtgagtcgtattaatttcgataagccaggttaa
cctgcattaatgaatcggccaacgcgcggggagaggcggtttgcgtattgggcgctcttccgcttcctcg
ctcactgactcgctgcgctcggtcgttcggctgcggcgagcggtatcagctcactcaaaggcggtaatac
ggttatccacagaatcaggggataacgcaggaaagaacatgtgagcaaaaggccagcaaaaggccaggaa
ccgtaaaaaggccgcgttgctggcgtttttccataggctccgcccccctgacgagcatcacaaaaatcga
cgctcaagtcagaggtggcgaaacccgacaggactataaagataccaggcgtttccccctggaagctccc
tcgtgcgctctcctgttccgaccctgccgcttaccggatacctgtccgcctttctcccttcgggaagcgt
ggcgctttctcaatgctcacgctgtaggtatctcagttcggtgtaggtcgttcgctccaagctgggctgt
gtgcacgaaccccccgttcagcccgaccgctgcgccttatccggtaactatcgtcttgagtccaacccgg
taagacacgacttatcgccactggcagcagccactggtaacaggattagcagagcgaggtatgtaggcgg
tgctacagagttcttgaagtggtggcctaactacggctacactagaaggacagtatttggtatctgcgct
ctgctgaagccagttaccttcggaaaaagagttggtagctcttgatccggcaaacaaaccaccgctggta
gcggtggtttttttgtttgcaagcagcagattacgcgcagaaaaaaaggatctcaagaagatcctttgat
cttttctacggggtctgacgctcagtggaacgaaaactcacgttaagggattttggtcatgagattatca
aaaaggatcttcacctagatccttttaaattaaaaatgaagttttaaatcaatctaaagtatatatgagt
aaacttggtctgacagttaccaatgcttaatcagtgaggcacctatctcagcgatctgtctatttcgttc
atccatagttgcctgactccccgtcgtgtagataactacgatacgggagggcttaccatctggccccagt
gctgcaatgataccgcgagacccacgctcaccggctccagatttatcagcaataaaccagccagccggaa
gggccgagcgcagaagtggtcctgcaactttatccgcctccatccagtctattaattgttgccgggaagc
tagagtaagtagttcgccagttaatagtttgcgcaacgttgttgccattgctacaggcatcgtggtgtca
cgctcgtcgtttggtatggcttcattcagctccggttcccaacgatcaaggcgagttacatgatcccca
tgttgtgcaaaaaagcggttagctccttcggtcctccgatcgttgtcagaagtaagttggccgcagtgtt
atcactcatggttatggcagcactgcataattctcttactgtcatgccatccgtaagatgcttttctgtg
actggtgagtactcaaccaagtcattctgagaatagtgtatgcggcgaccgagttgctcttgcccggcgt
caatacgggataataccgcgccacatagcagaactttaaaagtgctcatcattggaaaacgttcttcggg
gcgaaaactctcaaggatcttaccgctgttgagatccagttcgatgtaacccactcgtgcacccaactga
tcttcagcatcttttactttcaccagcgtttctgggtgagcaaaaacaggaaggcaaaatgccgcaaaaa
agggaataagggcgacacggaaatgttgaatactcatactcttcctttttcaatattattgaagcattta
tcagggttattgtctcatgagcggatacatatttgaatgtatttagaaaaataaacaaataggggttccg
cgcacatttccccgaaaagtgccacctgacgtctaagaaaccattattatcatgacattaacctataaaa
ataggcgtatcacgaggcccttacgtattaattaa
