# Supplementary material for: The Cis-Regulatory Code for Kelch-like 21/30 Specific Expression in Ciona robusta Sensory Organs
Source: Front Cell Dev Biol. 2020 Sep 11;8:569601. doi: 10.3389/fcell.2020.569601 (PMC7517041; doi:10.3389/fcell.2020.569601)
Supplement: TABLE S3 — (A) List of oligonucleotides used for functional assays. Names contain indication on the position of the target sequence inside the selected exon (e.g., Mitf-ex3 106 indicates that the target sequence is within exon3 starting from its nucleotide 106). In bold and capital letters the sgRNA N (19) target sequences. In lowercase the protospacer sequence (for OSO PCR) appended 3′ to a forward primer and, in reverse complement, appended 3′ to the reverse primer (Gandhi et al., 2017). In red are highlighted the oligos whose sgRNAs were able to cause deletions. “Specificity” indicates the capability of the synthetized sgRNA to target a specific sequence (0–100); “efficacy” represents a prediction of sgRNA ability to induce Cas9-mediated DSBs (0–100); “SNPs” is the number of potential single-nucleotide polimorphisms associated to each sgRNA, which can possibly affect its pairing to the target. All these values were retrieved from CRISPOR portal. (B) List of “peakshift” genomic oligos (in blue) used to verify the effect of deletions on specific exons (ex i.e., exon). [file Table_3.DOCX]

**Supplemental Table S3.** A) List of oligonucleotides used for functional assays. Names contain indication on the position of the target sequence inside the selected exon (e.g. Mitf-ex3 106 indicates that the target sequence is within exon3 starting from its nucleotide 106). In bold and capital letters the sgRNA N (19) target sequences. In lowercase the protospacer sequence (for OSO PCR ) appended 3′ to a forward primer and, in reverse complement, appended 3′ to the reverse primer [(Gandhi et al. 2017)](https://paperpile.com/c/PKxvT4/TmSF). In red are highlighted the oligos whose sgRNAs were able to cause deletions. “Specificity” indicates the capability of the synthetized sgRNA to target a specific sequence (0-100); “efficacy” represents a prediction of sgRNA ability to induce Cas9-mediated DSBs (0-100); “SNPs” is the number of potential single-nucleotide polimorphisms associated to each sgRNA, which can possibly affect its pairing to the target. All these values were retrieved from CRISPOR portal.

B) List of “peakshift” genomic oligos (in blue) used to verify the effect of deletions on specific exons (ex i.e. exon).

| **A** |  | | |  |  |  |
| --- | --- | --- | --- | --- | --- | --- |
| **Name** | **Primer sequence** | | | **Specificity** | **Efficacy** | **SNPs** |
| Mitf-ex3 106 rev Forward | g**CTTGTACAGGTAGTTGGGG**gtttaagagctatgctggaaacag | | | 99 | 62 | 1 |
| Mitf-ex3 106 rev Reverse | **CCCCAACTACCTGTACAAG**catctataccatcggatgccttc | | | 99 | 62 | 1 |
| Mitf-ex4 104 rev Forward | g**CGAAGGGTTGAATAGCTGG**gtttaagagctatgctggaaacag | | | 100 | 66 | 0 |
| Mitf-ex4 104 rev Reverse | **CCAGCTATTCAACCCTTCG**catctataccatcggatgccttc | | | 100 | 66 | 0 |
| Mitf-ex4 125 Forward | g**AAAGCGCTCCCACCAACGC**gtttaagagctatgctggaaacag | | | 100 | 64 | 0 |
| Mitf-ex4 125 reverse | **GCGTTGGTGGGAGCGCTTT**catctataccatcggatgccttc | | | 100 | 64 | 0 |
| Mitf-ex5 30 rev Forward | g**ACAAGTTGAGACGAGCTAG**gtttaagagctatgctggaaacag | | | 100 | 63 | 0 |
| Mitf-ex5 30 rev Reverse | **CTAGCTCGTCTCAACTTGT**catctataccatcggatgccttc | | | 100 | 63 | 0 |
| Mitf-ex5 39 rev Forward | g**GACGAGCTAGAGGACTCGC**gtttaagagctatgctggaaacag | | | 100 | 62 | 0 |
| Mitf-ex5 39 rev Reverse | **GCGAGTCCTCTAGCTCGTC**catctataccatcggatgccttc | | | 100 | 62 | 0 |
| Dmrt-ex1 30 For Forward | g**CCGGGCATAATAATTACCC**gtttaagagctatgctggaaacag | | | 100 | 67 | 0 |
| Dmrt-ex1 30 For Reverse | **GGGTAATTATTATGCCCGG**catctataccatcggatgccttc | | | 100 | 67 | 0 |
| Dmrt-ex1 54 rev Forward | g**AAATAAAGCCGCCGCTGCG**gtttaagagctatgctggaaacag | | | 100 | 64 | 0 |
| Dmrt-ex1 54 rev Reverse | **CGCAGCGGCGGCTTTATTT**catctataccatcggatgccttc | | | 100 | 64 | 0 |
| Dmrt-ex1 62 for Forward | g**GCGGTCTCAAACCCCGCAG**gtttaagagctatgctggaaacag | | | 100 | 64 | 0 |
| Dmrt-ex1 62 for Reverse | **CTGCGGGGTTTGAGACCGC**catctataccatcggatgccttc | | | 100 | 64 | 0 |
| Dmrt-ex2 76 for Forward | g**TGTTGTACGGTGCTACACA**gtttaagagctatgctggaaacag | | | 100 | 62 | 0 |
| Dmrt-ex2 76 for Reverse | **TGTGTAGCACCGTACAACA**catctataccatcggatgccttc | | | 100 | 62 | 0 |
| Dmrt-ex2 64 for Forward | g**GTGAATTAAGGCTGTTGTA**gtttaagagctatgctggaaacag | | | 99 | 60 | 0 |
| Dmrt-ex2 64 for Reverse | **TACAACAGCCTTAATTCAC**catctataccatcggatgccttc | | | 99 | 60 | 0 |
| Dmrt-ex3 56 rev Forward | g**GACGCCATGTCCTGAGCAG**gtttaagagctatgctggaaacag | | | 100 | 62 | 0 |
| Dmrt-ex3 56 rev Reverse | **CTGCTCAGGACATGGCGTC**catctataccatcggatgccttc | | | 100 | 62 | 0 |
| Dmrt-ex3 57 rev Forward | g**AGACGCCATGTCCTGAGCA**gtttaagagctatgctggaaacag | | | 100 | 60 | 0 |
| Dmrt-ex3 57 rev Reverse | **TGCTCAGGACATGGCGTCT**catctataccatcggatgccttc | | | 100 | 60 | 0 |
| Msx-ex3 209 rev Forward | g**CTGTGACGTCAGACCTGGT**gtttaagagctatgctggaaacag | | | 99 | 73 | 0 |
| Msx-ex3 209 rev Reverse | **ACCAGGTCTGACGTCACAG**catctataccatcggatgccttc | | | 99 | 73 | 0 |
| Msx-ex2 38 Forward | g**CAAACCTGAAAGAAAAACC**gtttaagagctatgctggaaacag | | | 94 | 63 | 0 |
| Msx-ex2 38 reverse | **GGTTTTTCTTTCAGGTTTG**catctataccatcggatgccttc | | | 94 | 63 | 0 |
| Msx-ex1 64 Forward | g**GTGACGTCACCAAAACCTA**gtttaagagctatgctggaaacag | | | 98 | 61 | 0 |
| Msx-ex1 64 Reverse | **TAGGTTTTGGTGACGTCAC**catctataccatcggatgccttc | | | 98 | 61 | 0 |
| **Control oligos** |  | | |  |  |  |
| Mesp.5 Forward | g**GGTAAACGGTCGAACCTGTG**gtttaagagctatgctggaaacag | | | 100 | 70 | 0 |
| Mesp.5 Reverse | **CACAGGTTCGACCGTTTACCC**catctataccatcggatgccttc | | | 100 | 70 | 0 |
|  | |  |  |  |  |  |
| **B** | |  |  |  |  |  |
| **Peakshift Oligos** | |  |  |  |  |  |
| Mitf ex3 Forward | | ACGAAACGTTTAAACTACTAGG |  |  |  |  |
| Mitf ex3 Reverse | | AAATCGTTTGTAGTACTAATTCAAAC |  |  |  |  |
| Mitf ex4 Forward | | TTTTTCCTACAGGTTGAGACTG |  |  |  |  |
| Mitf ex5 Reverse | | GGCGTAAAATATAGTTAGTCGACG |  |  |  |  |
| Dmrt ex1 Forward | | ACAGTTCGTTTAACTGGCATG |  |  |  |  |
| Dmrt ex1 Reverse | | CCATGACTCGTTGTCGTTC |  |  |  |  |
| Dmrt ex2 Forward | | AGGATATCCATATTAACAGGTTGC |  |  |  |  |
| Dmrt ex2 Reverse | | ATTTACCTGTCGGCGATGTC |  |  |  |  |
| Dmrt ex3 Forward | | ATGCGTCATAATGTTGCAGG |  |  |  |  |
| Dmrt ex3 Reverse | | CTGGCCTTCTGGTTTCATC |  |  |  |  |
| Msx ex2 Forward | | TGCGTTGCAGTAAACGAATC |  |  |  |  |
| Msx ex3 Reverse | | AACGCAGAGTTTTACTTACGG |  |  |  |  |
